# Investigating a Metrical Hebb Effect for lists of words

**DOI:** 10.1177/17470218241285884

**Published:** 2024-11-14

**Authors:** Andrew W. Paice, Andrew J. Johnson, Rebecca Legg, Eleonore Smalle, Michael P.A. Page

**Affiliations:** 1 University of Hertfordshire, Hatfield, UK; 2 Bournemouth University, UK; 3 Ghent University, Ghent, Belgium; 4 Tilburg University, Tilburg, Netherlands

**Keywords:** Hebb Repetition Effect, metrical patterns, working memory, immediate serial recall

## Abstract

In four experiments, we describe the first finding of a Metrical Hebb Effect. Participants are shown to exhibit a Hebb Repetition Effect for repeating list-wide stress patterns across sequences of familiar words, even though the lexical items within the “repeating” lists do not themselves repeat. Experiment 1 established the presence of a Hebb effect for metrical patterns, demonstrating significant learning of list-wide metrical patterns over successive presentations. Experiment 2 investigated the effect’s longevity, showing the persistence of learned metrical information after a spacing of three non-repeating lists. Experiment 3 revealed that the effect did not persist over a longer spacing of eight intervening lists. Experiment 4 investigated the learning mechanism, suggesting that chunking, rather than item-position binding, might account for the observed learning of metrical patterns. The authors propose that metrical-pattern learning represents a process of gradual integration of sequences of weak and strong stress accents into higher-level units representing the stress patterns within, and across, words. We briefly discuss some implications of the Metrical Hebb Effect for phonological word-form learning and for speech perception and production.

The working memory model (WMM), as first described by Baddeley and Hitch (1974) and as celebrated in this special issue, represents a key strand in the history of cognitive psychology, specifically the cognitive psychology of memory. The concept of the phonological loop, that was for a long time the most closely studied of the WMM components, has offered crucial insights into the nature of verbal short-term memory and has motivated a very large body of detailed theorizing and experimental research. In the mid-1990s, one of the authors of this article (MP) worked closely with Alan Baddeley and others (notably Dennis Norris and Rik Henson) to develop computational models of immediate serial recall (ISR) for verbal materials, and we focussed on phenomena such as the phonological similarity effect, the word length effect, and the irrelevant sound effect. This research, and other closely related work by Graham Hitch and Neil Burgess, resulted in three competing models (Burgess & Hitch, 1992, 1999; Henson, 1998; Page & Norris, 1998) that took their place alongside others with similar scope (e.g., Brown, Neath & Chater, 2007; Brown, Preece, & Hulme, 2000; Farrell & Lewandowsky, 2002; Nairne, 1990; Neath & Nairne, 1995; etc.). In parallel with these modelling efforts, Baddeley, Gathercole, and Papagno (1998) significantly clarified the theoretical importance of the phonological loop by making a strong case that it is intimately involved in the learning of phonological word forms. This general hypothesis motivated at least some groups to extend their computational models to include, within their scope, the transfer of serial-order memory from short term to long term. In this regard, Page and Hitch, working on a BBSRC grant with Norris, Cumming, and McNeil, concentrated on the Hebb Repetition Effect (Hebb, 1961), making the case, on the basis of a number of experimental lines (see below), that the Hebb Repetition Effect was a laboratory analogue of word-form learning. This work culminated in the extended neural network models of Burgess and Hitch (2006) and Page and Norris (2009), both of which attempted to model aspects of the Hebb Repetition Effect. The latter model specifically did so by positing a chunking process that could be distinguished from a potential alternative mechanism involving the strengthening of position-item associations, a simple version of which had been contraindicated by early experiments (Cumming, Page, & Norris, 2003). The years since these models emerged have seen the Hebb Effect become of key interest to modellers of serial-order memory, and a brief summary of some of the associated research is offered below.

As has been the case throughout the 50-year history of the WMM in relation to other effects, theory and experiment in relation to the Hebb Effect have developed in parallel, with a fruitful and ongoing dialogue between the two. In this spirit, the research presented here extends work on the Hebb Effect in a new direction, examining the potential learning of the metrical information implicit in verbal sequences alongside, but distinct from, the more familiar learning of their phonological/lexical content. As such, we see this work as honouring the legacy of the Baddeley and Hitch (1974) working memory model, acknowledging that model’s fundamental importance as a theory of short-term memory while exploring its wider relevance to theories of word learning, speech perception, and speech production.

## Introduction

Word learning is a complex process requiring the successful integration of semantic, phonological, and orthographic representations. The phonological information (i.e., the sounds that comprise a given word) is also intimately linked with a prosodic level of information, as both unravel, in step, over time. However, unlike the semantics, orthography, or phonology of a word, the underlying prosodic information is limited in its variety. For example, the prosodic information relating to metrical stress is limited to the classification of accents as either weak or strong, and the number of patterns one can generate is further constrained by the regularities of metrical patterns within a given language (as discussed later). An overview of the key aspects of prosodic information will be provided first, focussing on the role of stress. We will then present an overview of research on the Hebb Effect before introducing the main topic of the paper: an investigation into whether representations of *metrical patterns* are learned by a process similar to that by which representations of word forms are learned.

### Prosody

Prosodic information relates to the rhythm and intonation present, or perceived, in an acoustic stream. It is often defined as *suprasegmental*—that is, it is conceived at a level above that of the constituent vowels and consonants that comprise a given word (Cole, 2015; Cutler & Jesse, 2021). Although definitions and use might vary, prosodic information encompasses the effects of changing pitch, syllable duration, and stress. Here, our work primarily focuses on the last of these—that is, on the way in which patterns of weak and strong stresses form a *metrical pattern* that unravels over the course of the perception or production of a word or sequence of words. To be precise, these patterns, formed of weak and strong stresses, are often described as involving increased vowel intensity and duration (Laver, 1994; Odden, 2005; Plag, Kunter, & Schramm, 2011) and can involve the alteration of pitch and of emphasis (Gordon & Roettger, 2017; Zahner, Kutscheid, & Braun, 2019). For example, in two-syllable words, an iambic stress pattern (weak-STRONG) characterises the word “implore,” and a trochaic stress pattern (STRONG-weak) characterises the word “donkey.” Moreover, in three-syllable words, you can have dactylic stress patterns (STRONG-weak-weak, e.g., “Appetite”), anapaestic stress patterns (weak-weak-STRONG, e.g., “comprehend”), amphibrachic stress patterns (weak-STRONG-weak, e.g., “condition”), and so on. Metrical information reflects the stress-structure of a given portion of speech or of a piece of music; it is one observable structure through which prosodic information is realised. It is the pattern of stresses that unravels over a word or a sentence, and here, for our purposes, it is the descriptor for the pattern of stresses that span the length of a list of words in our experiment.

Regarding prosody in general, research indicates that, from birth, there is a sensitivity to prosodic information, at least to rhythm (Mehler et al., 1988), that can be seen, for example, in a neonatal infant’s ability to discriminate between language rhythms (Ramus, Nespor & Mehler, 1999). At nine months of age, this ability expands to include the awareness of prosodic information such as stress patterns (Jusczyk et al., 1992), which, along with the processing of pauses and syllable durations, allows for words to begin to be segmented from the acoustic stream (Cutler, 1994; Johnson & Jusczyk, 2001; Langus et al., 2017). Stresses, which Doelling et al. (2014) described as “acoustic edges,” or a heightening of speech amplitude, aid this segmentation of the lexical components of the speech stream. Therefore, the ability to process prosodic information can be viewed as a pre-lexical process that assists in the segmentation and understanding of lexical information. Evidence suggests that the usefulness of stress as a lexical marker is appreciated implicitly. This is seen in the intuitive use of infant-directed speech (ID-speech), where adults alter their speech when speaking to infants by exaggerating stress patterns, lengthening syllables, and otherwise manipulating the dynamics of their speech (Kuppen & Bourke, 2017). Infants appear to prefer ID-speech (Cooper & Aslin, 1994), and it appears to facilitate segmentation of the acoustic stream (Leong, Kalashnikova, Burnham, & Goswami, 2014).

Focusing on *metrical patterns* and stress, it appears that their use in English (and in Dutch) often highlights the presence of word boundaries (Cutler & Carter, 1987). Infants appear better to notice words with trochaic (STRONG-weak) stress patterns as opposed to iambic (weak-STRONG) patterns. The preponderance of trochaic words is a statistical feature of the English speech these children were hearing (Jusczyk, Houston & Newsome, 1999). Stress placement may perform a similar role in helping to locate the word boundaries in other languages. For example, in French, a stressed syllable often indicates the end of a word, and in Polish, it is the penultimate syllable that is often stressed. These regularities provide prosodic cues to the word boundaries present in the acoustic stream. Event-related potential (ERP) research, by Böcker, Bastiaansen, Vroomen, Brunia, and Gelder (1999), provided support for this idea by showing a relationship between sensitivity to metrical stress and speech segmentation skill. Van Donselarr, Koster, and Cutler (2005) also noted that this process of using prosodic information from the speech signal is continuous, involving ongoing updates to the process of identifying word boundaries.

It is clear, therefore, that the role of prosodic information is crucial across many aspects of language learning, primarily as some formative, pre-lexical processes in infants but also in the moment-by-moment analysis and computation of a speech stream in adulthood, allowing, in both cases, for enhanced segmentation of words from the acoustic stream. This suggests a degree to which prosodic information facilitates lexical access (Cutler & Norris, 1988). Soto-Faraco et al. (2001) provide evidence for how, in Spanish, lexical stress performs a similar role in lexical access as does segmental content. Van Donselaar, Koster, and Cutler (2005) showed that when the stress pattern of a word was replaced with a different stress pattern (one compatible with a different word), then identification of the word was slowed, indicating that some inhibition was caused by the stress mismatch. Additionally, Cutler and Van Donselaar (2001) showed that stress placement can remove the effect of lexical competition, indicating again that the pattern of stresses directly affects lexical activation. Lastly, Cooper, Cutler, and Wales (2002) showed that English and Dutch listeners do indeed use stress in the recognising of words.

Some research has also focused on the role of stress in the grouping of sounds. The idea of grouping fits in directly with the research described previously regarding the role of stress in the segmenting of the acoustic stream. Prosody, in general, does appear to play a role in grouping. For example, Frankish (1989) proposed that within a grouped list of items, the pauses between items behave like pauses in everyday speech. These pauses can highlight where groupings should best be made, with the process of grouping enhancing recall. Beyond mere grouping effects, though, how might different metrical patterns become established as distinct mental representations in the first place? The work described below attempts to investigate some mechanisms that might underpin their learning.

### Hebb Effect and word learning

Over the last twenty years, growing research has implicated the Hebb Repetition Effect (HRE) as a laboratory analogue of word-form learning (Mosse & Jarrold, 2008; Norris, Page, & Hall, 2018; Saint-Aubin & Guérard, 2018; Szmalec et al., 2009; Szmalec, Page, Duyck, 2012). In the original experiments by Hebb (1961), participants were presented with lists of nine digits and tasked with recalling those nine digits in the correct order (an immediate serial recall, ISR, task). Participants were unaware that one of the lists was repeatedly presented, every third list. Performance on this repeating list improved as a function of repetition. This performance increase on the repeating list occurs regardless of participants’ reported awareness of the repetition of this list over the course of an experiment (McKelvie, 1987; Stadler, 1993; though see Musfeld, Souza & Oberauer, 2023). The effect is often described as an archetypal task in which information can be seen to pass from short-term to long-term memory. There have been numerous models of immediate serial recall (including Brown et al., 2000, 2007; Burgess & Hitch, 1992, 1999; Farrell & Lewandowsky, 2002; Gupta, 2008; Henson, 1998; Neath, 2000; Neath & Brown, 2006; Page & Norris, 1998) and some (notably Burgess & Hitch, 2006; Page & Norris, 2009) have been extended to attempt to model the HRE, as noted previously. We also know that the HRE has been seen across modalities, including with visual stimuli (Johnson & Miles, 2009; Johnson & Miles, 2019; Page et al., 2013), auditory stimuli (St-Louis, Hughes, Saint-Aubin, & Tremblay, 2019; Szmalec, Page & Duyck, 2012), spatial stimuli (Tremblay & Saint-Aubin, 2009; Couture & Tremblay, 2006), faces (Horton, Hay, & Smyth, 2008), and even olfactory stimuli (Johnson, Cauchi, & Miles, 2013; Johnson, Shaw, and Miles, 2016). Given that the Hebb Effect is a well-established and ubiquitous effect in serial-order learning, it is not unreasonable to think that a Hebb Repetition Effect might occur for rhythmic/metrical patterns too, possibly associated with the learning of phonological word forms.

Evidence for the Hebb Repetition Effect as a laboratory analogue for word learning began with correlational evidence (Mosse & Jarrold, 2008), followed by a number of papers looking at experimental evidence for this hypothesis. Page and Norris (2009) give the example of learning a list of letters such as “B-J-F-M-L” and posited that the mechanism by which this is learnt during an ISR/Hebb task is analogous to that which would be needed to learn the word “Bejayeffemelle” in a naturalistic setting. They noted that the HRE is fast and long-lasting, like word-form learning (Cumming et al., 2003); many Hebb lists can be learned simultaneously, much like one might expect words to need to be learned in parallel (Saint-Aubin et al., 2015); children show a Hebb effect (Smalle et al. 2016, Smalle et al., 2018), as one might expect if the process is related to word learning; and Hebb repetition learning occurs at various spacings, just as word learning must (Page et al., 2013). The role of the HRE as a laboratory analogue of vocabulary learning, focuses, for the ISR of speech-based materials at least, on the word form (i.e., the sequence of sounds that comprise a given word). However, as described previously, prosodic information is intimately involved in the learning, understanding, and production of words. Therefore, we sought to use the HRE paradigm to investigate how stress patterns are established over the course of pattern repetitions and whether any learning resembles what is seen in the HRE literature. To summarise our results, we do find that a Hebb-like repetition effect occurs for *metrical patterns*, though we show that this is a relatively short-lived effect and one that is, like other previously established Hebb effects, more compatible with a chunking-based account than a simple positional one.

## Experiment 1

### Introduction

In Experiment 1, we sought to establish whether there is a Hebb Effect for metrical information. Experiment 1 serves as the paradigm from which the subsequent experiments follow, both in their methodology and in their statistical analysis. The experiment deploys an immediate serial recall (ISR) task for which participants first listen to a list of words. Then these words are re-presented on the screen, in a random array, and participants are tasked with clicking on the words in the order in which they had heard them. Note, therefore, that we have an auditory presentation of a word list, with a visual representation at recall.

### Methodology

#### Participants

A total of 174 participants were recruited via the online recruitment platform Prolific, and participants were paid £4.50 for 30 minutes of their time. There were two preregistered exclusion criteria applied to the participants, as data were collected: first, we excluded participants whose error rate was 65% or more; and second, we excluded participants whose error rate was 15% or less. The former criterion controlled for individuals who did not appear to be sufficiently engaged in the task, and the latter criterion controlled for individuals who were high performers and who were, therefore, unlikely to show the learning benefit associated with the Hebb effect (owing to ceiling effects). These error rates were computed on the first presentation of each *repeating metrical pattern list* and on all the lists with non-repeating metrical patterns.

Additionally, any participant who took part in the experiment was prescreened, via Prolific, ensuring participants were between 18 and 50 years of age (inclusive) with English as their first language. Through a process of testing and excluding participants based on these preregistered exclusion criteria, we finished data collection once 120 participants could be included in the final dataset.

The target of 120 participants was decided upon by a power analysis via simulation. Although unsure of an exact effect size for our proposed Metrical Hebb Effect, we were able to use pilot data from previous Hebb repetition paradigms as a starting point. Using the “simr” and “lme4” packages in “R,” a generalised linear mixed effects model (GLMM) was fit to data from 500 simulations. The simulated data used the parameters harvested from models fitted to data from previous research. The GLMM had a single fixed effect of list type (i.e., the categorical variable: [1] repeating list and [2] non-repeating list, explained in more detail in the following sections), and the observed effect size was lowered slightly for the simulations, giving us the opportunity to detect a smaller Hebb effect than is common. The analysis revealed that 120 participants were sufficient to achieve 90% power to detect such an effect.

#### Materials

Generation of materials required the creation of *metrical patterns*, which were used to generate *lists* of these *metrical patterns* (which we call *metrical pattern lists*), these were then populated with items from the *word lists*. The experimental trials were specific orderings of these *metrical pattern lists*. Additionally, the lists were represented auditorily and, therefore, recordings of each word were made. The following section details the specifics of this process.

#### Metrical patterns

Six *metrical patterns* were decided upon, referred to here as A1, A2, B1, B2, C1, and C2. The *metrical patterns* are as shown in Table 1 (where “*w*” refers to a weakly stressed syllable and “***S***” refers to a strongly stressed syllable).

**Table 1.** Metrical patterns with stress patterns and example words.

Metrical pattern	Stress pattern	Example word (orthography)	Example word (IPA)
A1*	S	Blue	/bluː/
A2*	S	Camp	/kæmp/
B1	w-**S**-w	Condition	/kənˈdɪʃən/
B2	**S**-w-w	Attitude	/ˈætɪtjuːd/
C1	w-**S**-w-w	Capacity	/kəˈpæsɪti/
C2	w-w-**S**-w	Democratic	/ˌdɛməˈkrætɪk/

* A1 and A2 have an identical stress pattern: just a single strong accent.

The choice of single-, three-, and four-syllabled words allows for variation in the grouping of these patterns. Furthermore, more variation was allowed in the three- and four-syllabled words as there were two variations for each length, depending on where stress is placed within these words. The inclusion of two metrical variations of words with the same number of syllables would make it such that, should an effect be found, a word-length effect would be an insufficient explanation of any observed effects.

#### Metrical pattern lists

The six *metrical patterns* mentioned were then pseudo-randomly grouped into 15, generic *metrical pattern lists* (see Appendix A). An example of a *metrical pattern list* is C2, A1, B1, B2, A2, C1. The pseudo-random nature of the ordering of these lists ensured that across the fifteen *metrical pattern lists*, the same *metrical pattern* did not appear in the same position more than twice. Therefore, we mitigated against any confounding effects of potential pattern-position learning across lists as an explanation for any within-list learning.

#### Word sets

Using the six *metrical patterns*, 10 words were selected that matched the stress pattern of a given *metrical pattern*. For example, for the *metrical pattern* B1, which is characterized by the stress pattern *w-* ***S****-w*, the following words were selected:

B1: “Arrangement,” “Condition,” “Consider,” “Distinction,” “Electric,” “Establish,” “Foundation,” “Instruction,” “Location,” “Position”

Therefore, within a given *metrical pattern list* the *metrical patterns* could be populated with one of 10 words, sampled across the 10 presentations without repetition. The word sets can be found in Appendix B.

#### Experimental trials

From the 15 *metrical pattern lists*, 10 were randomly chosen to be used as *repeating metrical pattern lists*, and the other five acted as *non-repeating metrical pattern lists*. Across the presentations of a *repeating metrical pattern list*, all the words from the associated word set were sampled. Importantly, therefore, only the stress pattern repeated across the presentations of a given *repeating metrical pattern list*, meaning that the words (i.e., the phonological content) were always different. It is the *metrical pattern* that repeats across “repeating” lists in this version of the Hebb Effect, not the words themselves. This is, therefore, to our knowledge, the first time a verbal Hebb effect has been studied for lists in which the words themselves do not repeat.

#### Audio files

Each word was recorded by a single male voice. Recordings were sampled at 44 kHz in a sound booth designed to prevent acoustic reverberation. Words were recorded in time to a visual (flashing) metronome, one after the other, in a continuous take so that the single male speaker naturally P-centred each word. Audio files were manipulated using Audacity and Praat. The best examples of each word were selected based on a subjective decision of how accurately they sounded like the target word, inspection of the spectrogram, and the absence of auditory clicks and other miscellaneous noise. Each word, regardless of length, was represented by an audio file of the same length, arranged such that the P-centre occurred approximately 600 ms into the file. This P-centring ensured that words would sound natural when the audio files were concatenated in different orders into various lists. Note that the P-centre, or perceptual centre, of an auditorily presented word refers to its “psychological moment of occurrence” as defined by Morton, Marcus, and Frankish (1976). The P-centre does not strictly align with the middle of the acoustic signal of a given spoken word or with its onset. Word sequences will only sound regular if their P-centres are evenly distributed in time, as was ensured by our procedure.

#### Design and procedure

Participants engaged in an immediate serial recall (ISR) task, where items were presented auditorily one after the other. Words were presented at a rate of one word every 1200 ms. After hearing all the words in a given list, the words from that list appeared on the screen. Participants had been instructed to click the words on the screen in the order in which they had heard them. Once a word was clicked, it disappeared so that no single word could be clicked twice. Participants responded to 60 such lists.

The experiment contained six blocks. In each of the first five blocks, there were ten lists presented: nine lists had the same underlying *metrical pattern* across each entire list, which we call the *repeating metrical pattern list*, and one list, which we call the *non-repeating metrical pattern list*, had a different (and unique) *metrical pattern*. The *repeating metrical pattern list* varied by block and was counterbalanced by participant so that a given participant heard blocks involving five different *repeating metrical pattern lists*, and, among all participants, all ten *repeating metrical pattern lists* were heard in the different block positions.

Additionally, we had another manipulation alongside “repeating” and “non-repeating” trials, which was the *position* of the non-repeating list within the block. As stated previously, there were nine *repeating metrical pattern lists* and one *non-repeating metrical pattern list* per block; the placement of this single *non-repeating metrical pattern list* varied. Either it appeared in the final position of a given block, or it appeared in the penultimate position within a given block. That is to say, in a given block, participants were either presented with nine *repeating-metrical-pattern lists* and then the final list was the *non-repeating metrical pattern*, or, alternatively, participants were presented with eight *repeating metrical pattern lists*, the *non-repeating metrical pattern list*, and then a final *repeating metrical pattern list*. Manipulating the placement of this *non-repeating metrical pattern list* allowed us to investigate whether any effect we might find across the *repeating metrical pattern lists* was simply due to a very short-term effect carrying over from one list to the next rather than a wider, more complex and hypothesized process of Hebb repetition learning.

**Figure 1.**
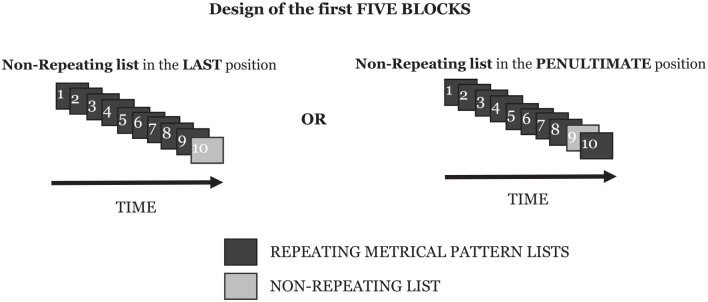
The design that Block 1 to Block 5 followed, showing the two levels of the condition by which the placement of the non-repeating list within a block was manipulated.

**Figure 2.**
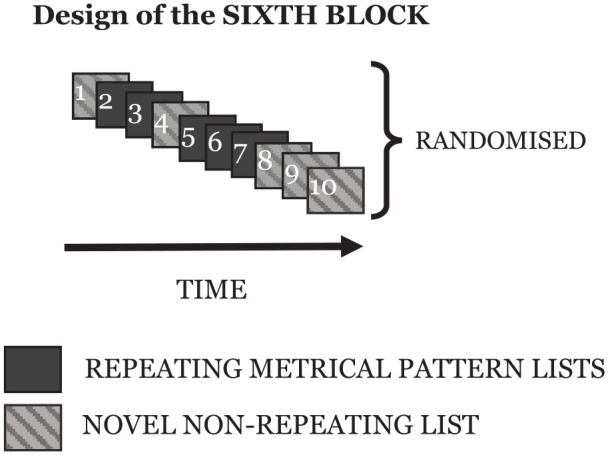
The design of the sixth block for each participant. Note that the order of the repeating and novel non-repeating lists was randomised. The repeating metrical pattern lists comprise one novel list for each of the five repeating metrical patterns that had been used, one per block, in the first five blocks.

The sixth block differed from the first five as it contained one re-presentation of each of the *repeating metrical patterns* from the previous five blocks, together with a set of five novel *non-repeating metrical pattern lists*. This final block allowed for some analysis relating to the longevity of any putative learning. However, it proved to be a less sensitive measure than hoped (discussed later) and generated the further investigations embodied in the other experiments presented in this paper. The design of each of the first five blocks, and the final sixth block, are shown in Figure 1 and Figure 2.

### Results

Data were collected via the Gorilla experimental website, and recruitment was facilitated by the use of Prolific. The models that comprise the statistical analysis are presented in the order in which they were preregistered on the Open Science Framework. We attempted, at all stages, to adhere to the preregistered analysis, however, where deviations do occur, they will be noted. All datasets can be found on the OSF platform for this project: https://osf.io/hzgkr/.

#### Descriptive statistics

##### The reduction in errors over presentation

To establish the plausibility of a Metrical Hebb Effect, it was important to see whether, over successive presentations in a block, there was a reduction in the errors on the *repeating metrical pattern lists*. The experiment had sixty ISR trials in total; each trial contains the auditory presentation of a single word-list and the immediate serial recall of that list. These 60 lists are separated into six blocks, and each block has 10 trials. As stated previously, each of the first five blocks contained nine *repeating metrical pattern lists* and a single *non-repeating metrical pattern list*. A different underlying *repeating metrical pattern* was used for each block, and these were counterbalanced across all participants.

**Figure 3.**
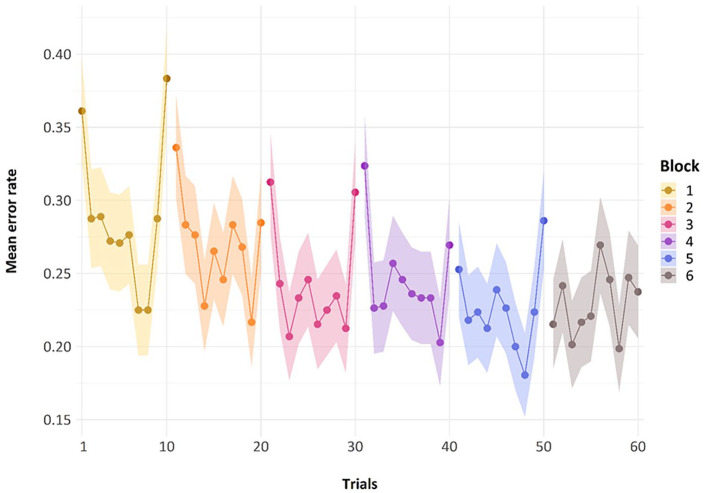
Mean error rate for each trial by block in Experiment 1. Error bars are two standard errors above and below the mean; they are calculated with the total observations, not the total subjects, to better reflect the variability in the data.

It did indeed appear that mean performance over the nine representations of a *repeating metrical pattern list* led to reduced errors within each block (see Figure 3). The first five blocks show a decrease in errors across the block, with the final point in each block here being the non-repeating list. As can be seen in Figure 3, errors on this final non-repeating list shoot up, giving performance on each block a jagged U-shape. Note that for this graph, the manipulation of whether this non-repeating list was in the final or penultimate place has been glossed over to simplify the graphic representation.

**Figure 4.**
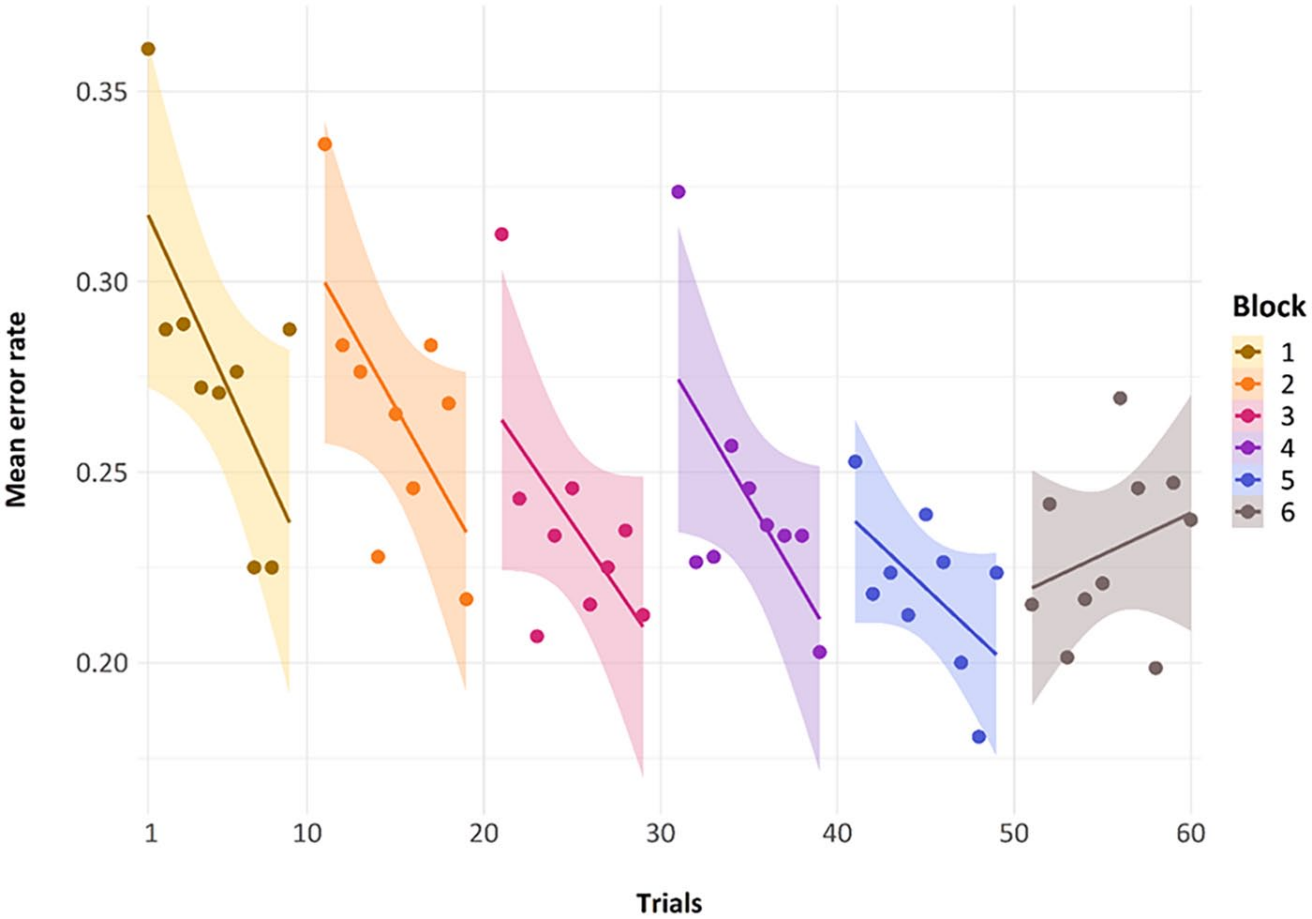
Mean error rate for each trial by block in Experiment 1, with linear regression lines.

The general trend of improvement across the presentations of the *repeating metrical pattern list* can also be seen in Figure 4. Here, we have removed the *non-repeating metrical pattern list* and show only the nine *repeating metrical pattern lists*. The addition of a regression line shows this general trend of reducing errors over presentations per block, for the first five blocks. The final block, wherein we present five new lists, one for each of the *repeating metrical patterns* from the previous five blocks and five novel *non-repeating metrical pattern lists* whose specific *metrical pattern* across a list has never been seen before, has a random scattering of data points, as we would expect because the placement of these novel and re-presentation lists are randomly presented across trials 51 through to 60, by participant. We will discuss performance in this block later.

**Figure 5.**
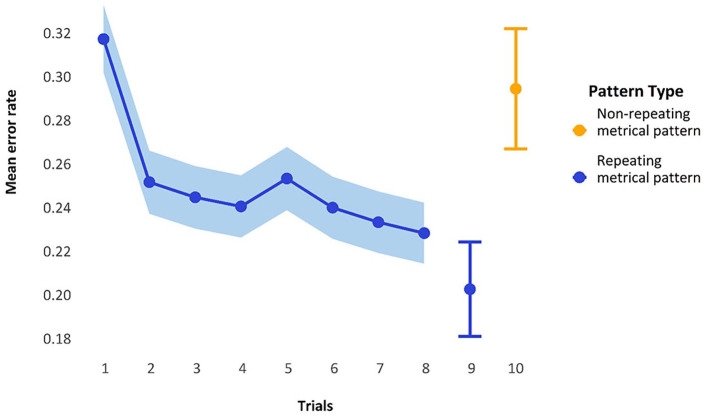
Mean error rate collapsed over block, showing performance over trials on repeating metrical pattern lists compared with the non-repeating metrical pattern lists. Trials 1 to 8 show SEs calculated by observations; trials 9 and 10 show the estimated marginal means and model-calculated SEs from the GLMM below.

Figure 5 displays the mean performance per trial-in-block when we collapse over the first five blocks. There is quite a large initial improvement in performance after the first trial, with a more gradual improvement toward the ninth presentation of the *repeating metrical pattern list*; the final point is the *non-repeating metrical pattern list*, which, as expected, elicits more errors than the preceding *repeating metrical pattern lists*.

##### The transposition matrix and the serial position curve, as compliance checks

**Figure 6.**
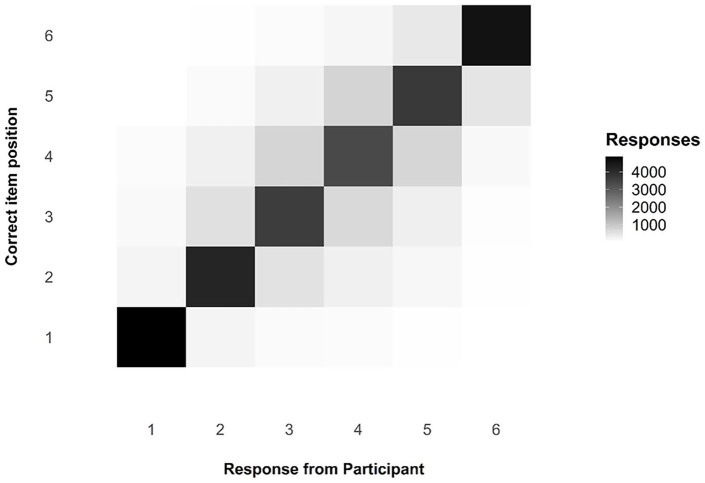
Transposition matrix as a shade map, comparing responses by participants to the actual position of any given item.

One question of interest when considering serial-order tasks is that of transpositions, which we can be observed via the transposition matrix below, visualised as a shade map (see Figure 6). Note that the diagonal line across the matrix indicates those responses that were in the correct position; the colour is very dark, indicating that responses were modally in the correct position. The distribution of shade around that middle section, moving from dark to light, indicates that, when errors were made, participants were often swapping items in the middle of the list with nearby items, as we would expect. These transposition errors are exceptionally common in ISR tasks and in Hebb Effect research, and the reproduction of this familiar pattern in this experiment is a useful compliance check for this online experiment.

**Figure 7.**
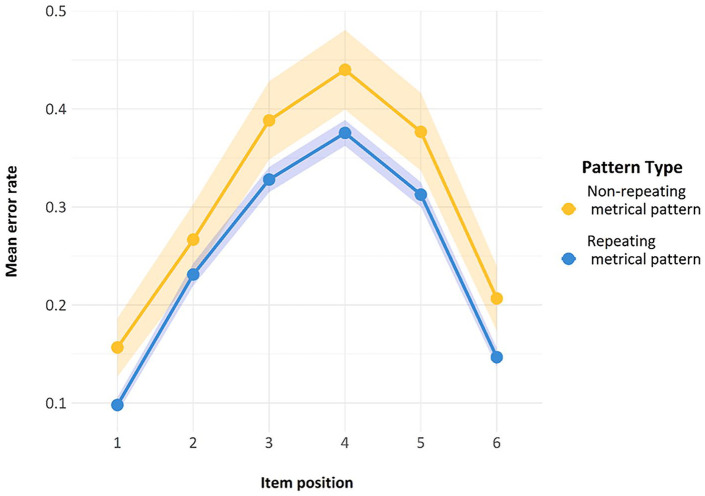
Serial position curve for the trials used in Model 1. Error bars are two standard errors above and below the mean; these are calculated with the total observations, not the total subjects.

Finally, we also looked at another standard feature of ISR research—namely, the serial position curve. As can be seen in Figure 7, both lines reproduce the classic serial position curve for auditory presentation. Errors are fewer in the initial and the final positions, representing a classic primacy and recency effect, with a slightly stronger primacy effect. The presence of this curve is indicative of the fact that the task was indeed being carried out as a serial recall task by participants and constitutes a second check on compliance.

#### Inferential statistics

All models were preregistered on the OSF website and are presented here in the order presented in the preregistration document. All models were conducted as generalised linear mixed effects models, which allowed us to model by-subject and by-item variance where appropriate. All analysis was conducted with a logit link function.

##### Model 1

To remind the reader of our primary hypothesis: we wanted to investigate the potential presence of a Hebb effect for metrical information. Within a given block, participants were presented with a set of *repeating metrical pattern lists*. These lists shared the same *metrical pattern* of stresses across the entire list; however, the words within these lists were different. Either at the end of a block, or in the penultimate place, a non-repeating list was presented, which did not share the same *metrical pattern* as any other list in that block. If there was a Hebb effect for the metrical information underpinning the repeating lists, we would expect that the final repeating list would elicit fewer errors than the non-repeating list, as individuals would have established some kind of representation of the order of the metrical structure in the repeating list. To be clear, we expect the final *repeating metrical pattern list* to have benefitted from all the previous presentations of this repeating *metrical pattern* and, therefore, elicit fewer errors than the nearby *non-repeating metrical pattern list*. Furthermore, we predicted that the position of this non-repeating list would not affect the results, as we presumed that if a Hebb effect were present then its effects would survive a single intervening non-repeating list.

##### Random effects structure

It is good practice when running GLMMs to attempt to fit the largest and most appropriate random effects structure within the model. In a typical Hebb paradigm, one can consider “subject” and “item” as random effects. In the following model, and all models that follow, the *lexical* “item” is not modelled; rather, we include the *metrical* “item” in the model. In other words, we do not model “item” at the level of phonology (e.g., “queen,” “addition”)—in statistical terms, that would have led to a design matrix that was too sparse. Instead, we model the underlying stress pattern of a given “item” (e.g., ***S***, *w-* ***S****-w*). The random effects structure was reduced so that the model converged and singularities were removed in a stepwise process where slopes with the lowest variance were removed first. For Model 1, the following random effects structure was used (using the common lme4 syntax):

(pattern_type|subject)+(1|metrical_pattern)

Therefore, we had a random intercept for both subject and *metrical pattern*, and a random slope for *pattern_type* by subject but not by *metrical_pattern*.

##### Fixed factors and outcome variables

The model contained two fixed factors. The first was *pattern type*, which had two levels: (1) *repeating metrical pattern list* and (2) *non-repeating metrical pattern list*. The second fixed factor was *position type*, which had two levels indicating the position in a given block of the *non-repeating metrical pattern list*, either (1) in the penultimate position of a block or (2) in the final position of a block. The outcome variable is binary, indicating whether or not the correct item was recalled in the correct position. The full model, presented here in the popular lme4 syntax, is as follows:

$$\begin{array}{l} \mathrm{error}\;\sim \;\mathrm{pattern\_type*position\_type}\;\\ \;\;\;\;\;\;\;\;\;\;\;\;\;\;\;\;+\;\left(\mathrm{pattern\_type|subject}\right)\;+\;\left(\mathrm{1|metrical\_pattern}\right) \end{array}$$

##### Results of Model 1

**Figure 8.**
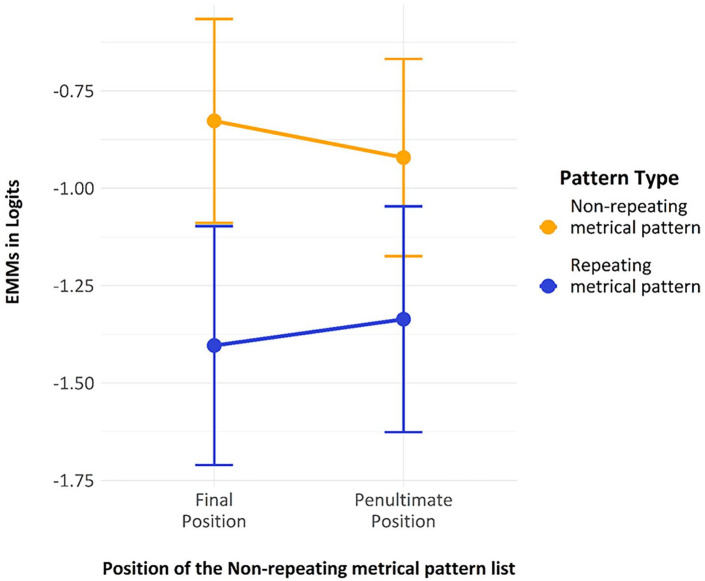
The estimated marginal means with error bars showing two standard errors, derived from Model 1.

The fixed factors were effect-coded so that the test could be interpreted much like a classic ANOVA, with main effects and an interaction. There was a significant main effect of “*pattern_type*”; *z* = −4.76, *p* < 0.001, one-tailed, indicating that errors on the final *repeating metrical pattern list* were significantly fewer than those for the *non-repeating metrical pattern list*. The main effect of “*position_type*” was not statistically reliable (*p* = 0.44), indicating that the learning of the *metrical pattern* survived a spacing of one *non-repeating metrical pattern list*. This suggests the effect is not just a list-to-next-list effect and suggests a more complex process is occurring. Finally, and perhaps not unexpectedly, the interaction between “*pattern_type*” and “*position_type*” was not significant (*p* = 0.96). Estimated marginal means from the model are shown in Figure 8.

In summary, the significant effect of *pattern type* is promising evidence for the presence of a Hebb effect for metrical information, and the lack of a significant effect of *position type*, suggests that the learning of this metrical information survives a spacing of at least one non-repeating list. Indeed, the lack of an interaction between *pattern type* and *position type* confirms that the benefit for the *repeating metrical pattern list* over the *non-repeating metrical pattern list* is not just a list-to-next-list effect.

We preregistered a follow-up to Model 1, labelled “model 2” in the preregistration; however, as Model 1 provides strong evidence for the presence of Hebb Effect for metrical information, and as this “Model 2” does not add a lot to the finding, we have moved the analysis to Appendix C.

##### Model 3

**Figure 9.**
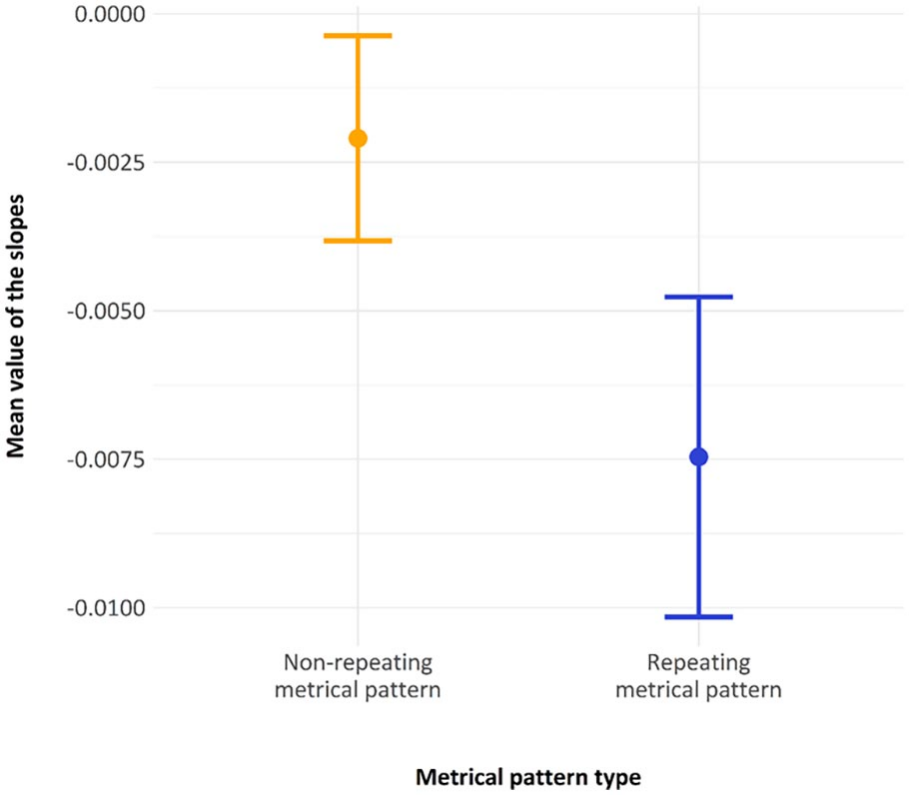
The derived slope across non-repeating metrical patterns (between blocks) and the mean slope across repeating metrical patterns in a block, with error bars showing two standard errors.

We stated in our preregistration that we would investigate the slope in recall error over presentations of the *repeating metrical pattern lists* (within each block) and the *non-repeating metrical pattern lists* (across blocks). If there were just a general practice effect across the whole experiment, then these slopes should be approximately the same. In line with the preregistration, we computed and subsequently compared the mean slope over the nine presentations of the repeating-lists within each block to the slope of the five fillers across blocks. In the preregistration, we detailed how this was a supplementary analysis, as there was some concern that the repeating-list slopes were computed over a different time frame and over many more observations. The mean value of the slopes for the *repeating metrical pattern list* condition was −0.007 (SD = 0.0147), and the mean value of the slopes for the *non-repeating metrical pattern list* conditions was −0.0021 (*SD* = 0.009), as shown in Figure 9. A Welch’s paired sample t-test (one-tailed, as preregistered) indicated that the difference between these two slopes was statistically reliable (*p* < 0.001). Therefore, the slope showing performance improvements across the *repeating metrical pattern lists* was reliably steeper than that across the *non-repeating metrical pattern lists*.

##### Model 4

The sixth block of Experiment 1 comprised a re-presentation of each *repeating metrical pattern* from the previous five blocks, using novel word lists, and five novel metrical *non-repeating pattern lists*. The 10 lists were presented to participants in a random order. We preregistered that we would compare performance between these five returning *repeating metrical pattern lists* and the five novel *non-repeating metrical pattern lists*. The data used were just those from the final block.

The model had the following structure:

$$\begin{array}{l} \mathrm{error}\;\sim \;\mathrm{pattern\_type}\;+\;\left(\mathrm{pattern\_type|subject}\right)\;\\ \;\;\;\;\;\;\;\;\;\;\;\;\;\;+\;\left(\mathrm{1|metrical}\;\mathrm{pattern}\right) \end{array}$$

*Pattern_type* had two levels: (1) *returning repeating metrical pattern list* and (2) *novel metrical pattern list*. There was no reliable difference between the two levels (*p* = 0.42). As we found what looked to be a Hebb effect for metrical patterns in the previous models, this finding was surprising. We would expect a Hebb effect to be long-lasting, or at least to survive over the course of a single, 30-minute experiment. Follow-up analysis indicated that there was not much control over where the *returning metrical pattern list* appeared in the block, which may have played a role if this Hebb effect is very short-lived. The sixth block would, however, have been well suited to finding a traditional, long-lasting Hebb Effect. The fact that we found no such effect suggested that the putative Metrical Hebb Effect was quite short-lived.

### Discussion

The previous results provide good evidence that there is indeed a Hebb, or Hebb-like, effect present for the *metrical patterns*. Participants were able to extract and learn the order of the stress patterns across a given list, even though the lexical information (the specific words) across those lists was changing. To our knowledge, this is the first demonstration of a Hebb-like effect, where there are different words in “repeated” lists.

Our first experiment raised a number of questions regarding the character of the Hebb(-like) effect seen with lists of repeating *metrical pattern*: Is the effect of the same character as other more standard Hebb effects? And how long does the Metrical Hebb Effect last? The following experiments, therefore, sought to investigate the character of the apparent Metrical Hebb Effect, starting with its longevity.

## Experiment 2

### Introduction

In Experiment 2, we sought to replicate the finding from Experiment 1—namely that a Metrical Hebb Effect is present, and then to extend this finding to see whether the effect survives a spacing of three *non-repeating metrical pattern lists*. The issue is of interest because the Hebb Effect is typically long-lasting and has been documented to remain for weeks, months (Page et al., 2013), or up to a year (Smalle et al., 2018), at least in the phonological domain. It is notable from Experiment 1, though, that the apparent Hebb effect seen there is comparatively weak: even for nine consecutive repetitions of the exact same cross-list *metrical pattern*, we saw less than a 1% improvement in error rate per repetition. We wanted to explore, therefore, whether the Metrical Hebb Effect apparently shown in Experiment 1 is as long-lasting as other Hebb effects in the phonological domain. Our initial analysis of the last block in Experiment 1 suggested that the Metrical Hebb Effect was not long-lasting. In Experiment 2, therefore, we investigated the longevity of any learned representation of the metrical information underpinning a given list. Specifically, we asked whether such representations survive a spacing of three lists. The methodology was a truncated version of that used in Experiment 1.

## Methodology

### Participants

A total of 431 (initial run: 251; revised run:180) participants were recruited via the online recruitment platform Prolific. Unfortunately, the initial run’s data had to be disregarded. There was an error in counterbalancing such that only one of the six potential variations of the experiment was ever shown to participants. This error only became evident during the analysis of the results but it meant that the experiment had departed from our preregistration. The data from the revised run are therefore used in the data analysis below. (Although not detailed here, conclusions based on the analysis of data from the initial run were entirely compatible with the conclusions drawn below.)

Participants were paid £1.20 for 8 minutes of their time. Through a process of testing and excluding participants, based on the preregistered exclusion criteria, we finished data collection once 120 participants could be included. This number of participants had been suggested by a power analysis by simulation (see above), giving 90% power to detect an effect of the size seen in the equivalent portion of Experiment 1. Prescreening, via Prolific, ensured participants were aged between 18–50 (inclusive) with English as their first language. The preregistration for this experiment, as with all experiments detailed here, can be found on the OSF repository (https://osf.io/hzgkr/). The results of this power analysis informed our decision to have 120 participants in Experiments 2, 3, and 4.

### Materials

All materials were a subset of those used in Experiment 1. For counterbalancing, we did not need to use all the *metrical pattern lists*, which had been generated for the first experiment, partly because Experiment 2 was much shorter than Experiment 1 in having only one Hebb Effect induction block. The selected *metrical pattern lists* were those in which the single-syllable words did not land in the last position, nor did both single-syllable words appear in adjacent positions. (We considered it possible that these properties might make a list distinctive enough to confer an advantage.) Lastly, we tried to select those *metrical pattern lists* that appeared, in Experiment 1, to elicit the greatest Hebb effect for *metrical patterns*. In total, 6 *metrical pattern lists* were taken from the original 15.

### Design and procedure

**Figure 10.**
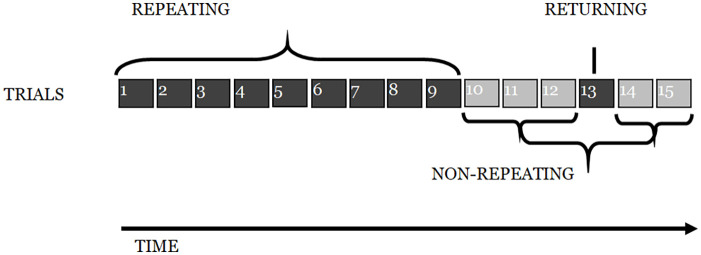
Depiction of the experimental design for Experiment 2.

As in Experiment 1, participants engaged in an ISR task, with 15 trials of a structure identical to that described previously: six words were presented auditorily at a rate of one word per 1200 ms, followed by a visual array of the stimulus words on which participants clicked with a mouse in what they took to be the correct order. The first nine lists were the *repeating metrical pattern lists* (i.e., they all had the same underlying *metrical pattern*); the next three were *non-repeating metrical pattern lists* (with unique patterns). The next list was another *repeating metrical pattern list* (i.e., with the same *metrical pattern* as the first nine lists), followed by two more non-repeating (unique) *metrical pattern lists* (see Figure 10). The experiment was designed to reveal whether there was the same learning of the underlying *metrical pattern* across the first nine lists, as seen in Experiment 1, and then whether the advantage in recalling such lists would survive the spacing of three non-repeating *metrical patterns* and result in enhanced performance for the returning *metrical pattern* (list 13, in Figure 10), relative to that for surrounding lists.

### Results

#### Descriptive statistics

**Figure 11.**
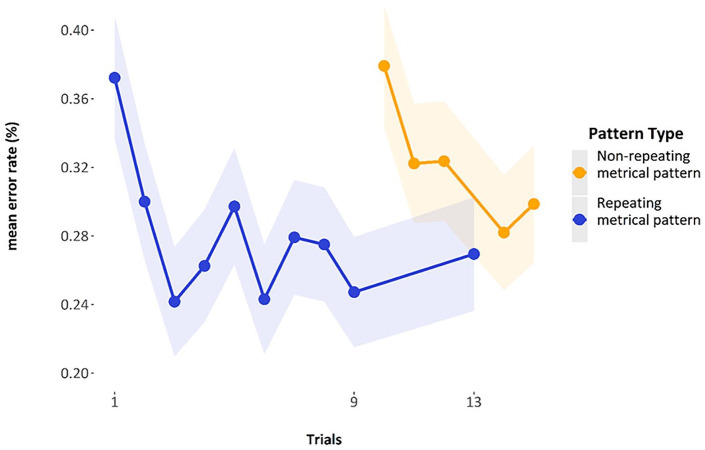
Mean error rates by trial, highlighting the final *repeating metrical pattern trial* (9th) and the return of the *repeating metrical pattern list* (13th). Error bars are two standard errors above and below the mean, and they are calculated with the total observations, not the total subjects.

The graph below (Figure 11) shows the mean error rate by trial, averaged over participants. The first *repeating metrical pattern list* has a mean of 37.2% (*SE* = 1.80%), and the final *repeating metrical pattern list* in that series has a mean of 24.7% (*SE* = 1.61%). When the *repeating metrical pattern list* returns it has a mean error rate of 26.9% (*SE* = 1.65%). The mean of the mean error rates for the *non-repeating metrical pattern lists* used in the analysis (i.e., the error rates of the 11th, 12th, 14th, and 15th list) was 30.67% (pooled *SE* = 1.70%).

##### Transposition gradients and serial position curves as compliance checks

**Figure 12.**
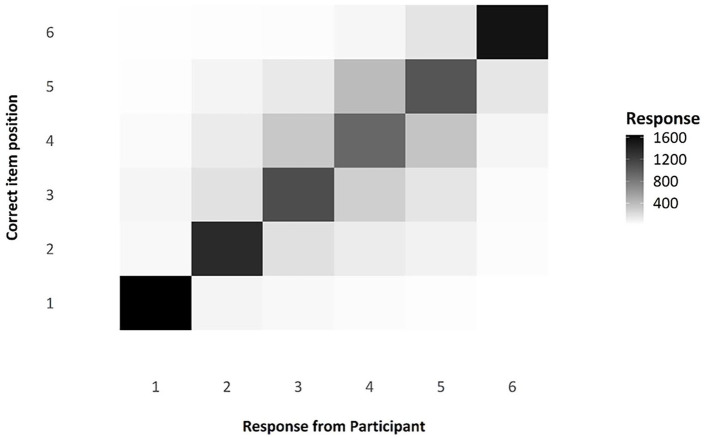
Transposition matrix as a shade map, comparing responses by participants to the actual position of any given item.

**Figure 13.**
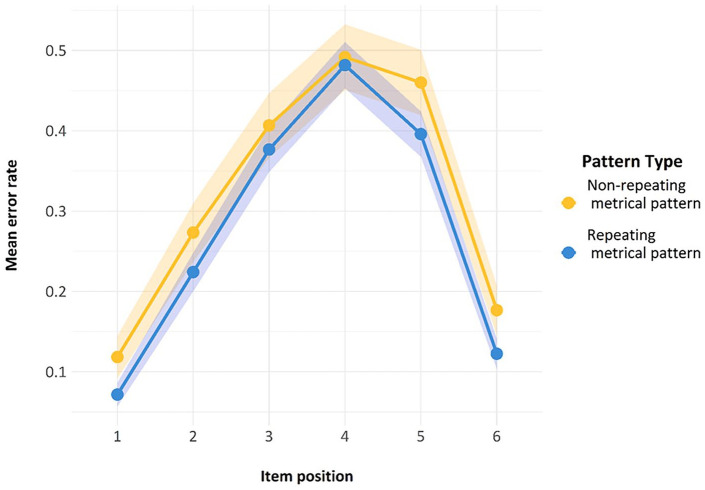
Serial position curves for the trials used in Model 1.

As a compliance check (see Experiment 1), we looked at the transposition gradients and serial position curves found in Experiment 2. With regard to transpositions, the shade map in Figure 12 shows that there are a high number of correctly recalled items, indicated by the darker shade on the leading diagonal, with errors largely comprising local transpositions. As you can see in Figure 13, there does indeed appear to be the characteristic inverted U-shape of a standard, auditory serial-position curve, with strong primacy and recency effects.

#### Inferential statistics

Two models were preregistered for Experiment 2. The details for the preregistration for this experiment can be found here https://osf.io/52v4f.

##### Model 1

The first model sought to replicate the finding from Experiment 1, namely that there is indeed a Hebb Effect for *metrical patterns*. In line with the preregistration, a generalised linear mixed effects model was generated in which we tested performance on the 9th presentation of the *repeating metrical pattern list* and compared this to the 10th list, which is the adjacent *non-repeating metrical pattern list*. If there is indeed a Hebb Effect for *metrical patterns*, we would expect that the *repeating metrical pattern list* would elicit fewer errors than the *non-repeating metrical pattern list*; this is because the *repeating metrical pattern list* should have benefitted from nine consecutive repetitions of the underlying *metrical pattern*.

##### Random effects structure

As in Experiment 1, we sought to fit the largest and most appropriate random effects model. However, these larger models failed to converge, so we removed slopes in a stepwise procedure, removing the slope that accounted for the lowest variance until there were no singularities and the model converged. The final random effects structure used was:

(pattern_type|subject)+(1|metrical_pattern)

##### Fixed effect and outcome variable

Model 1 had one fixed effect, *pattern type*, which had two levels: (1) *repeating metrical pattern list* or (2) *non-repeating metrical pattern list*. The outcome variable was binary, indicating whether participants had or had not recalled the correct item in a given position. As the only significant pattern of interest is one in which the *repeating metrical pattern list* outperforms the *non-repeating metrical pattern list*, the test was preregistered as one-tailed. The model was run with a logit link function. The final model, in lme4 syntax, was:

$$\begin{array}{l} \mathrm{Error}\;\sim \;\mathrm{pattern\_type}\;+\;\left(\mathrm{pattern\_type|subject}\right)\;\\ \;\;\;\;\;\;\;\;\;\;\;\;\;\;\;\;+\;\left(\mathrm{1|metrical\_pattern}\right) \end{array}$$

The fixed effect of *pattern type* was statistically reliable: *z* = 4.71, *p* < 0.001, one-tailed. Therefore, the final repeating list elicited fewer errors than the succeeding non-repeating list, offering evidence for the learning of the underlying *metrical pattern*. This is a replication of the finding from Experiment 1.

##### Model 2

The second model allowed us to investigate the longevity of any learning of the underlying metrical information. As with Model 1, we generated a generalised linear mixed effects model in line with our preregistered analysis. We took the performance on the *repeating metrical pattern list*, which returned after a spacing of three *non-repeating metrical pattern lists* (i.e., list number 13). We expected that the returning *repeating metrical pattern list*, despite the spacing of three *non-repeating metrical pattern lists*, would still elicit fewer errors than the surrounding *non-repeating metrical pattern lists*, assuming that learning from the first nine presentations of the *repeating metrical pattern lists* had established a representation of the order of the metrical information. Therefore, the test was preregistered as one-tailed, because the only result of interest is one in which the *repeating metrical pattern list* elicits fewer errors than the *non-repeating metrical pattern lists*. The data used are a subset of the main data, where we take performance on the 13th list (the returning *repeating metrical pattern list*) and compare it with performance averaged over the 11th, 12th, 14^th^, and 15th lists (the preceding and succeeding *non-repeating metrical pattern lists*).

##### Random effects structure

As with Model 1, the biggest and most appropriate random effects structure was generated, one in which *pattern type* was allowed to vary by subject and by item, and where subjects and items also had their own intercept (identical to the full random effects model first specified in Model 1). The random effects structure was reduced until singularities and convergence issues were no longer a concern. The resulting random effects structure was as follows:

(patterntype|subject)+(1|metrical_pattern)

##### Fixed effect and outcome variable

As in Model 1, the fixed effect of “*pattern type*,” was either (1) *repeating metrical pattern list* or (2) *non-repeating metrical pattern list*. Note that the *non-repeating metrical pattern list* condition therefore combined the performance of four lists, while the *repeating metrical pattern list* condition represented performance on a single list (list 13). The potential violation of the homogeneity of variance is less of an issue for mixed models. The outcome variable was binary, indicating whether or not the participant recalled the correct item in a given position. The final model (in lme4 syntax) was as follows:

$$\begin{array}{l} \mathrm{error}\;\mathrm{\sim pattern\_type}+\;\left(\mathrm{pattern\_type|subject}\right)\;\\ \;\;\;\;\;\;\;\;\;\;\;\;\;\;+\;\left(\mathrm{1|metrical\_pattern}\right) \end{array}$$

The fixed effect of “*pattern_type*” was statistically reliable: *z* = 2.44, *p* =0.007, one-tailed. Therefore, it does appear that there is a somewhat persistent advantage from the learning of *metrical patterns* over the initial nine presentations of the *repeating metrical pattern lists*. The advantage is evident even after three intervening *non-repeating metrical pattern lists*.

### Discussion

Experiment 2 was able to replicate the finding that the final *repeating metrical pattern list* elicits fewer recall errors than the subsequent *non-repeating metrical pattern list*, indicating that the repetition of the *metrical pattern* leads to some level of learning of that list-wide pattern of stresses. This is a direct replication of the effect seen in Experiment 1. We were also able to see that this learning appears to survive a spacing of three *non-repeating metrical pattern lists*. As part of our preregistration, we stated that, if we were to find a significant effect for three-spacing, we would then look to see if the advantage conferred by a repeated *metrical pattern* could survive a gap of eight intervening, non-repeating lists. This gave us another chance to replicate the presence of a Hebb Effect for *metrical patterns*, as shown in Experiment 1 and replicated here in Experiment 2.

## Experiment 3

### Introduction

Extending the finding of Experiment 2, that the Metrical Hebb Effect survives a spacing of three non-repeating lists, Experiment 3 had the spacing increase to eight non-repeating lists. The methods were identical to the previous experiment; the only change was the inclusion of additional lists to generate the required eight non-repeating lists between the penultimate and final presentation of the *repeating metrical pattern list*.

### Methodology

#### Participants

A total of 158 participants were recruited for Experiment 3 via the online recruitment platform Prolific. As Experiment 2 and Experiment 3 were covered in the same preregistration, we used the same exclusion criteria and power analysis as in Experiment 2. Participants were paid £1.30 for 9 minutes of their time. As preregistered, we stopped collecting data once 120 participants were able to be included in the experiment.

#### Materials

Materials were those used in Experiment 1. Eleven of the *metrical pattern lists* were needed for counterbalancing given that this was a much shorter experiment than Experiment 1 (that used fifteen *metrical pattern lists*) and slightly longer than Experiment 2 (that used six *metrical pattern lists*).

#### Design and procedure

**Figure 14.**
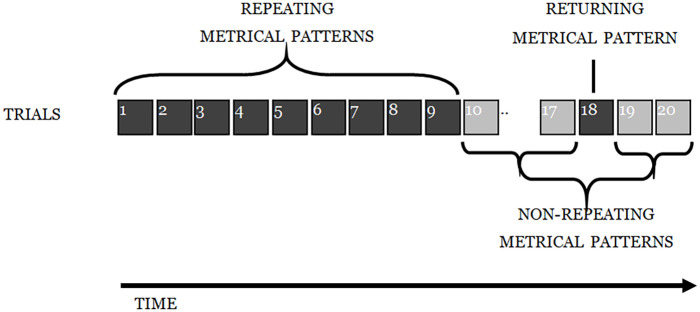
Depiction of the experimental design for Experiment 3.

The design was nearly identical to Experiment 2; however, participants had eight lists in between the initial nine presentations of the *repeating* *metrical pattern list* and the return for the final *repeating metrical pattern list* (see Figure 14).

### Results

#### Descriptive statistics

**Figure 15.**
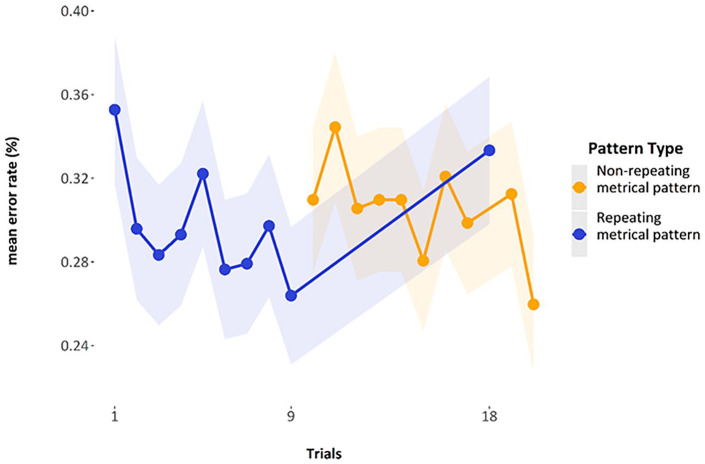
Mean error rates by trial, highlighting the final *repeating metrical pattern list* (9th) and the return of the *repeating metrical pattern list* (18th).

As for the data of Experiment 2, Figure 15 shows the mean error rate by trial, averaged over participants. The first *repeating metrical pattern list* had a mean of 35.3% error (*SE* = 1.78%), and the final *repeating metrical pattern list* in that series has a mean of 26.4% error (*SE*=1.64%); when the *repeating metrical pattern list* returns, it has a mean error rate of 33.3% (SE = 1.71%). The overall mean of the mean error rates for the *non-repeating metrical pattern lists* used in the analysis (i.e., the error rates of the 16th, 17th, 19th, and 20th list) was 29.8% (pooled *SE* = 1.7%).

##### Transposition gradients and serial position curves—compliance checks

**Figure 16.**
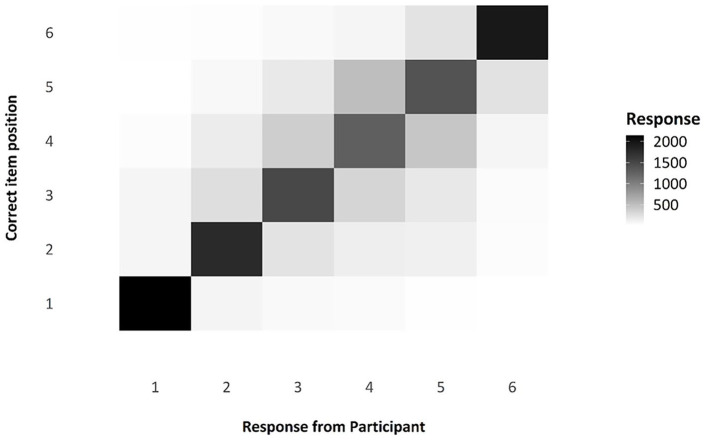
Transposition matrix as a shade map, comparing responses by participants to the actual position of any given item.

**Figure 17.**
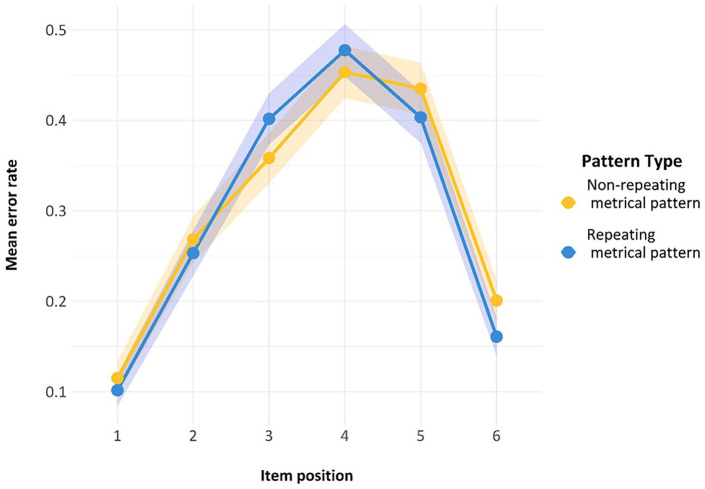
Serial position curves for the trials used in Model 1.

As with Experiments 1 and 2, the transposition shade map indicates that participants were correctly attending to, and engaging in, the ISR task, as demonstrated by most of the items being recalled in the correct position (dark shade on the diagonal), with the most likely error being the placement of an item in a position adjacent to its correct position (see Figure 16). The serial positive curves for Experiment 3 are also in line with what is expected for an ISR task—namely, the characteristic inverted U-shape caused by fewer errors at the start and end of a list (see Figure 17).

##### Inferential statistics

Experiment 2 and Experiment 3 were preregistered in the same document. We present the same analysis as that conducted in Experiment 2—namely, Model 1 will look at whether the ninth presentation of a *repeating metrical pattern list* elicits fewer errors than the four adjacent *non-repeating metrical pattern lists*.

##### Model 1

As stated previously, Model 1 investigates and attempts to replicate the presence of a Hebb Effect for metrical stimuli.

##### Random effects structure

The most appropriate and largest random effects structure for the model was fitted to the data, wherein the intercepts for both subject and metrical pattern, and slopes for the fixed effect of “*pattern_type*” by both subject and metrical pattern are included, resulting in a random effects structure that (in lme4 syntax) was as follows:

(pattern_type|subject)+(pattern_type|metrical_pattern)

##### Fixed effect and outcome variable

As in Model 1 for Experiment 2, there is just one fixed effect: “*pattern_type*,” which has two levels—(1) *repeating metrical pattern list* and (2) *non-repeating metrical pattern list*. The outcome variable was whether participants recalled the correct item in the correct place. The final model therefore looked as follows:

$$\begin{array}{l} \mathrm{Error}\;\mathrm{\sim pattern\_type}+\;\left(\mathrm{pattern\_type|subject}\right)\;\\ \;\;\;\;\;\;\;\;\;\;\;\;\;\;+\;\left(\mathrm{pattern\_type|metrical\_pattern}\right) \end{array}$$

The main effect of “*pattern_type*” was significant: *z* = 1.71, *p* = 0.04, one-tailed (as preregistered). The z-score was much smaller than that observed in Experiment 1 (*z* = 4.76) and Experiment 2 (z = 4.71), which implies that there was less learning in Experiment 3 than in the previous experiments.

##### Model 2

The second model would have investigated whether the metrical Hebb effect survives a spacing of eight intervening lists. The test was preregistered as one-tailed, because the only result of interest is one in which the *repeating metrical pattern list* elicited fewer errors than the *non-repeating metrical pattern lists*. However, looking at the means, we can see that the *repeating metrical pattern list* has a higher mean error rate (*m* = 33.3%, *SE* = 4.3%) than the overall mean of the error rates for the *non-repeating metrical pattern lists* used in the analysis (*m*= 29.8%, pooled *SE* = 4.17%). Therefore, as we proposed a one-tailed test, wherein we would only investigate the presence of an effect in which the *repeating metrical pattern list* elicited fewer errors than the *non-repeating metrical pattern list*, we can conclude that there was no effect to be found.

### Discussion

In summary, Experiment 3 aimed to further our understanding of the Metrical Hebb Effect, in particular, by examining how long this learning survives a set of intervening *non-repeating metrical pattern lists*. Model 1 revealed that the individuals did appear to show learning of the underlying *metrical pattern*, replicating the same finding from the previous two experiments, even though the z-value for this effect was much lower than in Experiment 1 and Experiment 2. Additionally, the results of Experiment 3 gave no evidence that the Metrical Hebb Effect could survive eight intervening *non-repeating metrical pattern lists*.

These findings show the Metrical Hebb Effect to be rather short-lived, in contrast to a traditional Hebb that is stable over weeks, months, or even years. For Experiment 3 specifically, the low levels of (albeit reliable) learning over the initial nine presentations of the *repeating metrical pattern list* were interesting. It does not seem likely, though, that this was the reason why we were unable to find the effect after eight intervening, *non-repeating metrical pattern lists*, given that the final *repeating metrical pattern list* was recalled numerically (though not reliably) worse than its *non-repeating metrical pattern list* controls.

## Experiment 4

Experiment 4 was used to investigate whether what we presumed to be a Hebb(-like) Effect, seen in our previous experiments, was in fact a consequence of *item-position* binding. In the Hebb Effect literature, some initial explanations focused on item-position binding. An item-position-binding account of the Hebb Effect would posit that the sequence ABCD is learned by binding A to a code representing the 1st list-position, B to the 2nd position, C to the 3rd position, and D to the 4th position. Positional accounts of the ISR task itself had been motivated by the finding, for example, that items that “protruded” from one trial to the next tended, more often than expected by chance, to appear in the same list position as that which they had originally been presented. Simple accounts, suggesting item-in-position learning as a mechanism of the Hebb Effect, were contradicted by Cumming, Page, and Norris (2003). Experiment 4 here addresses whether item-position binding is, however, an adequate explanation of the data from our first three experiments, or whether another explanation is needed. There was a concern that the single-syllable words, for instance, being quite distinct and recognisable, might have been easily bound to a particular list position, sufficient to cause the small recall advantage that we had observed.

To investigate this, Experiment 4 contained *transfer lists*, which helped us investigate the possible presence of item-position binding. Cumming, Page, and Norris (2003), using *transfer lists*, demonstrated that the Hebb Effect cannot be explained purely through a positional account. In these *transfer lists*, which were presented after a given list had been learned over repetitions (as per Hebb, 1961), Cumming et al. presented half of the list-items in the same positions as they had been presented in the previously learned Hebb-list, with the other half of the items each presented in a different position than in the learned Hebb-list. If serial order learning is purely positional, where positional codes are shared between lists (as was assumed to be the case based on the protrusion data), then those items held in the same position should have been recalled better than the items moved to different positions. However, Cumming, Page, and Norris (2003) showed that there was no performance difference between *same-position* items and the *different-position* items. Here, in Experiment 4, we used the same kind of *transfer lists* to reveal whether our apparent Metrical Hebb Effect is, in fact, just an instance of item-position binding or, more specifically, *metrical-pattern-to-position* binding.

### Methodology

#### Participants

A total of 210 participants were recruited via the online recruitment platform Prolific. Participants were paid £1.20 for 8 minutes of their time. Through a process of testing and excluding participants based on the preregistered exclusion criteria, we finished data collection once 120 participants could be included. Prescreening, via Prolific, ensured participants were aged between 18–50 (inclusive) with English as their first language. The preregistration can be found on the OSF research project page (https://osf.io/hzgkr/).

#### Materials

The materials used were identical to those used in Experiments 2 and 3, with the addition of the constructed *transfer lists* (see Table 2 for an example). A *transfer list* holds some *metrical patterns* within a list in the same position as they had occurred in the repeating (Hebb) list, while the others are reordered. Practically this means that either the odd or the even *metrical patterns* are held in the same position as they had been found in the *repeating metrical pattern list*. This even/odd manipulation occurred across subjects, as a given participant only saw one *transfer list* during the experiment. *Transfer lists* were created by taking the *repeating metrical pattern*, keeping three metrical patterns in the same position, and moving the other three metrical patterns. For example, if the odd-numbered items were kept in the same positions (1, 3, and 5) then the evens were mixed around to give a full list, such as 1,4,3,6,5,2 or, alternatively, 1,6,3,2,5,4.

**Table 2.** Example of an original list, and then the transformation into an “even” and an “odd” transfer list.

Initial order (metrical pattern)		Transfer list: even		Transfer list: odd	
RESOLUTION	(C2)	RADIATION	(C2)	BLUE	(A2)
EAST	(A1*)	NECESSITY	(C1)	COST	(A1)
POSITION	(B1)	ARRANGEMENT	(B1)	INDEPENDENCE	(C2)
CENTURY	(B2)	DANCE	(A1)	TELEPHONE	(B2)
WAIT	(A2*)	LOCK	(A2)	CONSIDER	(B1)
AVAILABLE	(C1)	CONFIDENCE	(B2)	COMBINATION	(C1)

* A1 and A2 have an identical stress pattern: just a single strong accent.

#### Design and procedure

**Figure 18.**
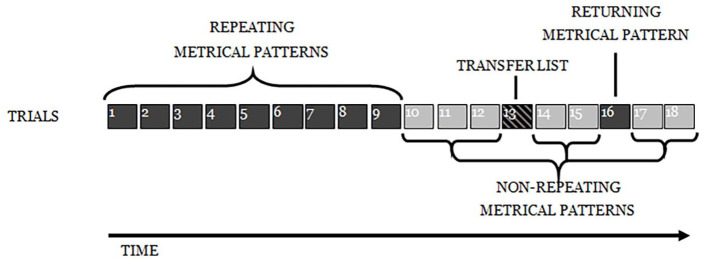
Depiction of the experimental design for Experiment 4.

The procedure was very similar to Experiments 2 and 3 (see Figure 18). An initial nine *repeating metrical pattern lists* were succeeded by three *non-repeating metrical pattern lists*, then the *transfer list*, then two more *non-repeating metrical pattern lists*, then a *repeating metrical pattern list*, and finally two *non-repeating metrical pattern lists*. The *non-repeating metrical pattern lists* here act to affect the required spacing and also serve as control lists for the error analysis.

The inclusion of the returning *repeating metrical pattern list* allowed us to see whether any learning that had occurred in the first nine lists could survive a gap of five non-repeating fillers and a *transfer list*. If there were no difference between same-position and different-position items on the *transfer list*, then it would have been impossible to know whether the null effect had been due to a genuine lack of position-item binding or simply due to no enduring learning of the *repeating metrical pattern* having occurred at all. With the inclusion of the returning *repeating metrical pattern*, we can see whether any lasting learning had occurred as a result of the first nine trials.

### Results

#### Descriptive statistics

**Figure 19.**
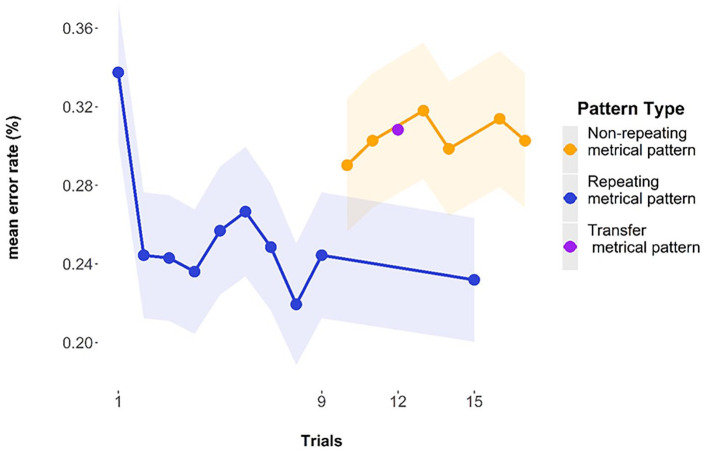
Mean error rate for the trials of Experiment 4. Error bars are 2 standard errors above and below the mean based on observations.

The general pattern of the data over trials is shown in Figure 19. As with the other experiments, we see a sharp decrease in the mean error rate over the first few presentations of the *repeating metrical pattern lists*. We can also see that there is a higher error rate for the *non-repeating metrical pattern lists* and the *transfer list*. Last, we can see that the final *repeating metrical pattern list*, which occurs after four other lists, has an error rate comparable to the later presentations of the *repeating metrical pattern list* in the induction block.

#### Transposition gradients and serial position curves—compliance checks

**Figure 20.**
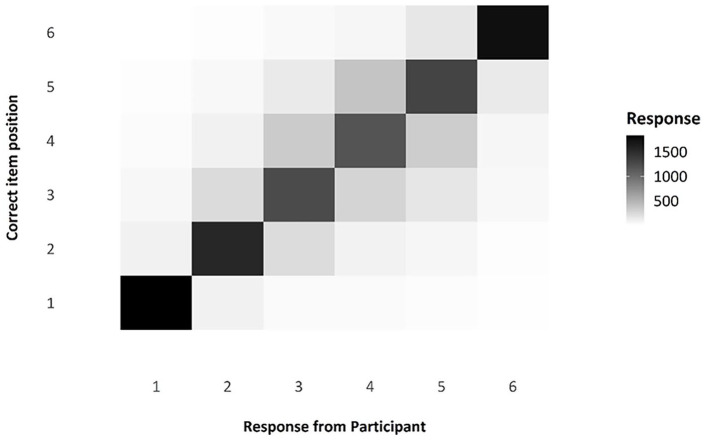
Transposition matrix as a shade map, comparing responses by participants to the actual position of any given item.

**Figure 21.**
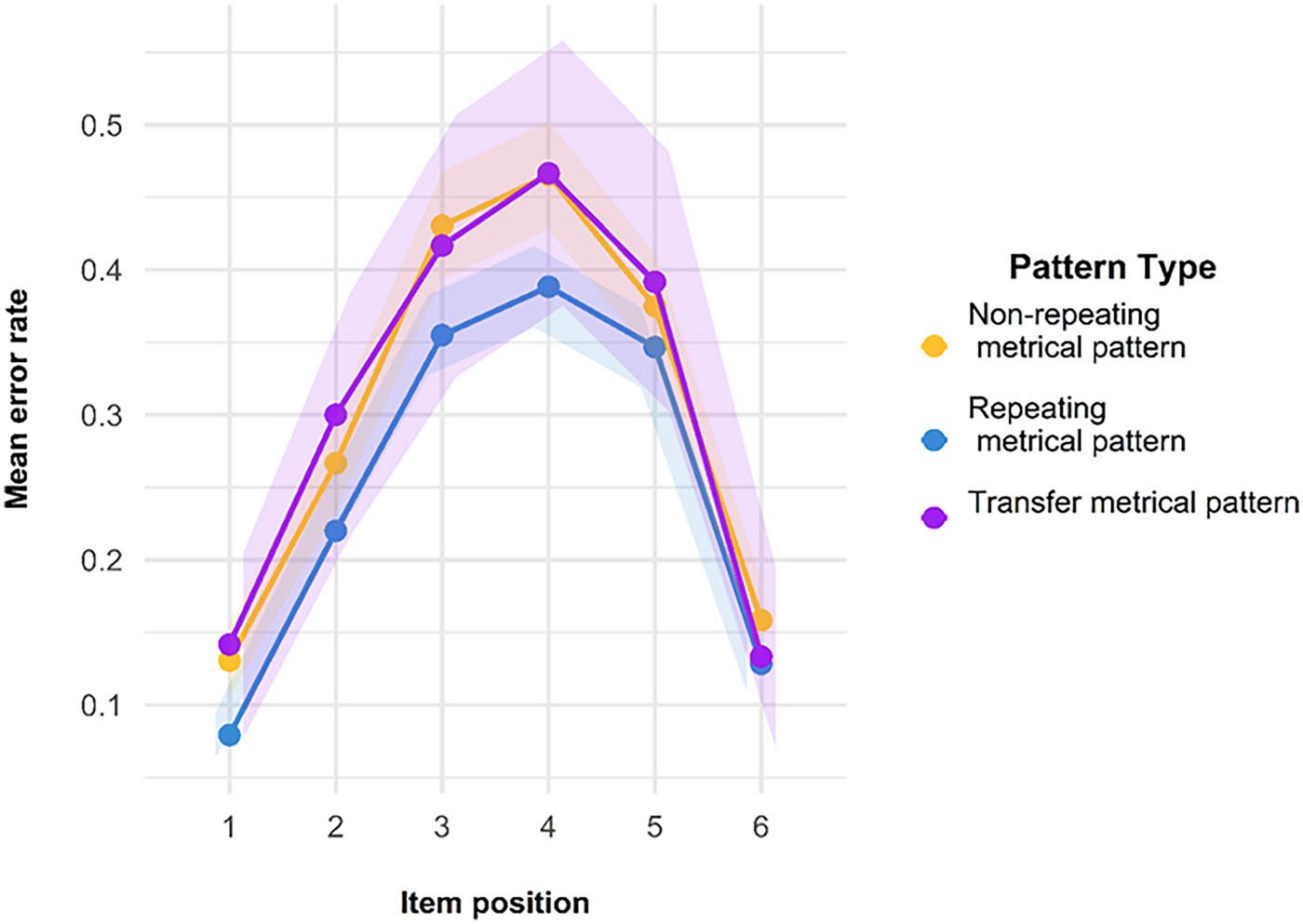
Serial position curves for all trials in Experiment 4, separated by pattern type.

As for the other experiments, the transposition gradients, as shown in Figure 20, are as we would expect if individuals were engaging in the ISR task correctly. The main diagonal is darker, indicating that most items were recalled in the correct position; positions in the middle of the list are lighter than end items, indicating more transposition errors. Where there are transpositions, items tend to be incorrectly recalled in a position adjacent to their correct position. Again, as for the other experiments, we checked to see whether there was a standard serial position curve present, as we would expect if participants had engaged in the ISR task as instructed. Figure 21 shows the serial position curves averaged over all of the presented lists. All these data are entirely consistent with participants’ having complied with the task instructions.

#### Inferential statistics

##### Model 1

As for each experiment, our first model sought to establish whether the initial presentations of nine *repeating metrical pattern lists* had led to a representation of the underlying *metrical pattern*’s being learned. This was tested by comparing the final two *repeating metrical pattern lists* with the first two *non-repeating metrical pattern lists*. In our previous experiment, we had noted that just taking performance on the final *repeating metrical pattern list* and comparing it with performance on the succeeding *non-repeating metrical pattern* was not a very sensitive measure of any effect, because performance could vary quite a lot over presentations. This preregistered change to our analysis sought to allow a Hebb Effect for metrical information to reveal itself more easily if such a pattern was indeed present. The model was therefore run on a subset of the data, where we took mean performance on the final two *repeating metrical pattern lists* and compared that with the mean performance on the first two *non-repeating metrical pattern lists*.

##### Random effects structure

The random effects structure was slightly larger than those that were able to be fitted previously, incorporating slopes and intercepts by “*pattern_type*” for both subject and items:

(pattern_type|subject)+(pattern_type|metrical_pattern)

##### Fixed effect and outcome variable

The model had a single fixed factor of “*pattern_type*,” which had two levels: (1) *repeating metrical pattern lists* and (2) *non-repeating metrical pattern lists*. These were coded such that there was no distinction between the 8^th^ and 9^th^ trial, nor between the 10^th^ and 11^th^ trial. The outcome variable was recall errors made, represented by a 1 for an item recalled in the correct place and a 0 for an item recalled incorrectly.

The mean error rate of the two *repeating metrical pattern lists* was 23.15% (*SE* = 1.57%), and mean error rate of the two proceeding *non-repeating metrical pattern lists* was 29.65% (*SE* = 1.7%) (see Figure 19). The model revealed that “*pattern_type*” had a reliable effect on recall, z= 2.13, *p* = 0.017, one-tailed (as preregistered). Therefore, as with all our experiments, it appears that across those nine presentations of the *repeating metrical pattern list*, some learning of the *repeating metrical pattern* underlying those lists was achieved.

##### Model 2

Model 2 was used to investigate whether overall performance on the *transfer list* differed from performance on the neighbouring *non-repeating metrical pattern lists*. Here, we used a subset of the data, where we took the two *non-repeating metrical pattern lists* before and the two after the *transfer list*, and compared the mean error rate on these four lists to the error rate on the *transfer list* itself. We would expect some difference to be present under an item-position account, with items kept in the same position tending to be recalled more accurately, and perhaps having a knock-on effect on other items too.

##### Random effects structure

The random effects structure was slightly smaller, as no model accounting for by-item variation was able to converge. The final random effects structure was as follows:

(pattern_type|subject)

##### Fixed effect and outcome variable

There was just one fixed-variable which was “*pattern_type*,” which had two levels: (1) *transfer list*, and (2) *non-repeating metrical pattern list*. The model revealed no reliable difference (*p* = 0.68) between the mean error rate on the *transfer list* (*M* = 30.2%, *SE* = 0.86%), and the mean error rate of the *non-repeating metrical pattern lists* (*M* = 30.8%, *SE* = 1.78%) (*see* Figure 20).

##### Model 3

Model 3 embodied the critical test of interest. If there is item-position binding, then items held in the same position should elicit fewer errors than items moved into different positions. The data used in this model were, therefore, only taken from within trial 13, the *transfer list*.

##### Random effects structure

The random effects structure was as follows:

(1|subject) + (1|metrical_pattern)

##### Fixed effect and outcome variable

**Figure 22.**
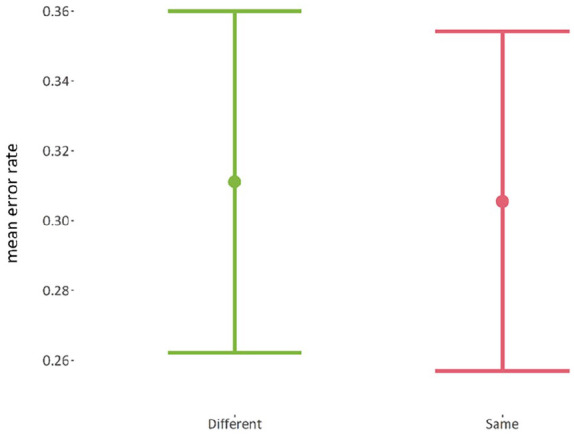
Mean error rate for the different-position items and the same-position items.

There was just one fixed-variable that was “*item_type*,” which has two levels: (1) same position and (2) different position. There was no reliable difference (*p* = 0.55) between the mean error rate of the *same-position* items (*M* = 31.1%, *SE* = 2.44%), and the mean error rate of the *different-position* items (*M* = 30.6%, *SE* = 2.43%). The lack of any effect is indicative of an account unfavourable to item-position learning. For a purely item-position account to be tenable, there ought to be some difference here between items retained and items not retained in position. By contrast, participants appear to recall those items held in the same position as in the repeating list at almost exactly the same level as they recall those items moved to different positions. The mean error rates for items held in the same position or represented in different positions can be seen in Figure 22.

##### Model 4

Model 4 was used to ensure that any null finding from Model 3 could not be explained by a simple lack of sufficiently enduring learning of the *repeating metrical pattern*. Therefore, the 15th list had a *repeating metrical pattern* identical to the initial nine representations, similar to how, in Experiment 2, the 13th list had the same *metrical pattern* as the first nine lists, and how in Experiment 3 the 18th list had the same *metrical pattern* as the initial nine lists. This *repeating metrical pattern list* had its performance compared with mean performance on the four closest *non-repeating metrical pattern lists*, that is, the two *non-repeating metrical pattern lists*, which preceded it and the two that succeeded it (i.e., the mean performance across the 13th,14th, 16th, and 17th lists).

##### Random effects structure

The random effects structure was:

(pattern_type|subject)+(pattern_type|metrical_pattern)

##### Fixed effect and outcome variable

There was just one fixed-variable which was “*pattern*_type,” which has two levels: (1) *repeating metrical pattern list* and (2) *non-repeating metrical pattern list*. The test revealed that the difference between the mean error rate of the *repeating metrical pattern list* (*M* = 23.2%, *SE* = 0.016%) and the mean error rate of the *non-repeating metrical pattern lists* (*M* = 30.8%, *SE* = 0.009) was significant: *z* = 3.86, *p* = 0.0002, one-tailed. Therefore, learning of the *repeating metrical pattern* appears to have survived a spacing of five *non-repeating metrical pattern lists* and a *transfer list*.

### Discussion

Experiment 4 once again showed that there was a Hebb-like effect for lists with a *repeating metrical pattern*. The last two *repeating metrical pattern lists* were recalled better than the two *non-repeating metrical pattern lists* that immediately followed them. This learning of the *repeated metrical pattern* survived a gap of five *non-repeating metrical pattern lists* and a *transfer list*, permitting improved performance when the *repeating metrical pattern* then returned. Performance on the intervening *transfer list* showed no recall advantage, though, either as a whole or specifically for words with metrical patterns that matched those in the same positions in the *repeating metrical pattern lists*. This suggests that the relatively weak learning that we see of the metrical pattern underlying a series of word-lists is not the consequence of some particular (maybe distinctive) metrical patterns being associated with particular list positions. Instead, the learning appears to be more consistent with the chunk learning that we have hypothesized for the Hebb Effect more generally (Page & Norris, 2009). In this case, it appears, we have evidence consistent with *metrical chunk* learning.

## General discussion

The results from the four experiments presented reveal the presence, and something of the nature, of a Metrical Hebb Effect. To our knowledge, this is the first instance of a reported Hebb Effect for verbal materials, where the list items (i.e., the words) change across trials, yet some underlying feature (here, the suprasegmental metrical information) is held constant.

Experiment 1 established the presence of a Hebb Effect for metrical information. We demonstrated that participants could learn list-wide *metrical patterns* over successive presentations, with a significant reduction in errors for *repeating metrical pattern lists* compared with *non-repeating metrical pattern lists*. Experiment 2 replicated this finding and investigated the longevity of the metrical Hebb Effect. The results confirmed the presence of a Hebb Effect for *repeating metrical pattern lists*, with learned metrical information surviving a spacing of three non-repeating lists. Experiment 3 extended the investigation into the longevity of the Metrical Hebb Effect by increasing the gap between the nine initial presentations of the *repeating metrical pattern lists* and the final *repeating metrical pattern list* to eight intervening *non-repeating metrical pattern lists*. The outcome indicated that the Metrical Hebb Effect observed in Experiments 1 and 2 did not persist over eight intervening non-repeating lists. This result highlighted the transient nature of the Metrical Hebb Effect and raised a question regarding whether it shared certain properties found for other, more familiar Hebb Effects. Experiment 4 aimed to discern whether the observed Hebb Effect for *repeating metrical pattern lists* could be attributed to item-position binding, a mechanism proposed in some accounts of serial-order learning. Using transfer lists in which we changed the positions of only some items within learned *repeating metrical pattern lists*, we found no significant difference in recall accuracy between metrical patterns maintained in the same position and those shifted to new positions. This outcome suggests that the learning observed in the previous experiments cannot be explained by a simple item-position binding mechanism for which list-position codes are hypothesized to generalize across lists. Instead, it implies a more complex process is at play, possibly involving the integration of *metrical patterns* into coherent, higher-level sequential structures.

It appears, to the authors at least, that the most parsimonious account of these data is that of *chunking*, whereby a gradual process of learning over repetitions leads to the establishment of a unitized representation of a list-wide metrical pattern, such that that representation subsequently *activates* on presentation of a list with that learned metrical pattern. The primacy model (Page & Norris, 1998) and subsequent papers linking the Hebb Effect and phonological word-form learning (Page & Norris, 2008, 2009) described word-form learning as a process by which previously uncommitted units become progressively better tuned and activated over successive presentations of a given, novel word form. Our experiments provide evidence for a similar process in the domain of metrical information. Over successive repetitions, an uncommitted unit (chunk unit) will come to represent the stress patterns implicit in a *repeating metrical pattern list*. We presume that there is a primacy gradient (to use the terminology of the primacy model) representing the serially ordered metrical information underlying a presented list, which might be activated in parallel with a different primacy gradient representing the lexical items in that list. In this context, the term chunking describes a gradual move from a given list’s being represented as a primacy gradient of activation across units representing, say, the individual metrical list-items to the same list’s being represented, after multiple repetitions, by the activation of a single chunk unit.

The weak and short-lived nature of the Metrical Hebb Effect, relative to other Hebb Effects, requires some explanation. We suggest two possibilities. The first is that there is a very narrow pool of potential *metrical patterns* possible (given the limited “vocabulary” from which list-items can be assembled, viz. strong and weak accents). Research suggests that the high overlap of items across repeated and non-repeated lists in a Hebb repetition paradigm detrimentally affects learning (Page et al., 2013). The second explanation is that the learning of extended, list-wide metrical patterns (i.e., of chunk units that would, potentially, respond to the whole of one of our *repeated metrical pattern lists*) is weak precisely because the lexical information is, by the design of our experiments, different for every one of the lists. It may be that lexical content is, in some sense, primary, with metrical content secondary. This would suggest, more generally, that a given metrical pattern (like, say, a *strong-weak-weak* pattern) is perhaps best learned over repetitions of a particular word with that metrical pattern (e.g., the word “agency”) rather than over the successive presentation of several different words, each with that particular metrical pattern. In other words, maybe a long-lasting metrical chunk is best (or even necessarily) learned as the secondary aspect of the learning of a long-lasting phonological chunk (i.e., a given word). The learning of an enduring metrical chunk might best accompany the learning of a *particular* word with that pattern, even if, once learned, it might then be attached to other lexical items sharing the same stress pattern. By extension, then, maybe a list-wide, repeating metrical pattern would be best learned if the sequence of lexical items in the list were also repeated.

We started this paper by discussing Baddeley et al.’s (1998) hypothesis that the phonological loop might best be thought of as a system for learning phonological word forms. The work presented above suggests that the metrical shape of a word might be learned in parallel, alongside its phonological content. Very briefly, why might this be? Two principal reasons suggest themselves. First, there are some words that have identical segmental (phoneme) content but whose meaning depends on the stress pattern applied. For example, the word “insult” is understood as a noun if stressed *strong-weak* but as a verb if stressed *weak-strong*. Disambiguating these two forms in continuous speech would require the recognition of the stress pattern sequence as well as, and in parallel with, recognition of the segmental sequence. Second, speech *production* (as opposed to perception) requires the generation of both segmental content and the appropriate corresponding stress pattern, if connected speech is to be accurately produced. It is no coincidence, therefore, that leading models of speech production (e.g., Dell & O’Seaghdha, 1992; Levelt, Roelofs, & Meyer, 1999) include separate chunk units expressing a word’s stress pattern (called *word-shape frame* or *metrical-shape* units, in the two models respectively). These stress-pattern chunks are very likely learned by exposure, and we suggest that our Metrical Hebb Effect is an experimental analogue of this learning process.

Finally, there is one corollary of our finding a Metrical Hebb Effect that potentially sheds light on an intriguing finding from an ostensibly different domain. The Metrical Hebb Effect confirmed previously strongly suggests that there is a short-term representation of the list-wide metrical pattern, formed every time a list of words is presented. It is not unreasonable, therefore, to conclude that that list-wide representation helps with the recall of the word-list itself. In an important paper on the word length effect, Hulme, Surprenant, Bireta, Stuart, and Neath (2004) showed that lists of mixed word length (i.e., lists containing one-syllable words alternating with longer words of three to five syllables) were recalled approximately as well as lists containing only short (one-syllable) words, even though pure lists of only long words were recalled much worse than pure lists of only short words. This finding was a challenge to various theories of the word-length effect, including to theories based on time-based decay. It is worth noting, therefore, that for a list purely consisting of single-syllable words, any short-term representation of the list-wide metrical pattern (such as the representation posited here) would be entirely undifferentiated and would, therefore, provide no information capable of assisting word-list recall. By contrast, for an alternating list of short and long words, any record of the list-wide metrical pattern would be of considerable help in constraining word-list recall—it would, at the very least, constrain the possible positions of the short words. If this additional constraint, aiding the recall of mixed lists relative to pure short lists, were approximately sufficient to offset any disadvantage caused by additional time-based decay for the mixed lists relative to the pure short, then that would offer a potential explanation for Hulme et al.’s influential finding.

In summary, we have expanded the conventional application of the HRE to metrical information, and our investigation demonstrates a brief yet perceptible repetition effect for the metrical patterns underpinning a list. Our findings indicate that metrical information displays swift but transient learning within this paradigm. This broadens the scope of the Hebb Repetition Effect and underscores its potential as an experimental tool for probing the evolution of different types of sequential representation over time.

## Supplemental Material

sj-docx-1-qjp-10.1177_17470218241285884 – Supplemental material for Investigating a Metrical Hebb Effect for lists of words Supplemental material, sj-docx-1-qjp-10.1177_17470218241285884 for Investigating a Metrical Hebb Effect for lists of words by Andrew W. Paice, Andrew J. Johnson, Rebecca Legg, Eleonore Smalle and Michael P.A. Page in Quarterly Journal of Experimental Psychology
