# Supplementary material for: Investigating a Metrical Hebb Effect for lists of words
Source: Q J Exp Psychol (Hove). 2024 Nov 14;78(2):284–309. doi: 10.1177/17470218241285884 (PMC11783982; doi:10.1177/17470218241285884)
Supplement: sj-docx-1-qjp-10.1177_17470218241285884 – Supplemental material for Investigating a Metrical Hebb Effect for lists of words [file sj-docx-1-qjp-10.1177_17470218241285884.docx]

Supplementary material for:

Investigating a Metrical Hebb Effect for lists of words

Andrew W. Paice. a

Andrew Johnson. b

Rebecca Legg. b

Eleonore Smalle. c, d

Michael P.A. Page*. a

1. *University of Hertfordshire*
2. *Bournemouth University*
3. *Ghent University*
4. *Tilburg University*

**corresponding author.* m.2.page@herts.ac.uk

#### Appendix A – The fifteen metrical patterns created for Experiment 1 (each code, A1, etc., represents a metrical pattern, such as Strong-Weak-Weak. See text for details)

|  |  | Position of the metrical pattern in the word list | | | | | |
| --- | --- | --- | --- | --- | --- | --- | --- |
|  |  | 1 | 2 | 3 | 4 | 5 | 6 |
| Repeating Metrical Patterns | 1 | C1 | C2 | A1 | A2 | B2 | B1 |
|  | 2 | A1 | A2 | C2 | B1 | B2 | C1 |
|  | 3 | C2 | A1 | B1 | B2 | A2 | C1 |
|  | 4 | C1 | A1 | A2 | B1 | C2 | B2 |
|  | 5 | A2 | C1 | A1 | B2 | B1 | C2 |
|  | 6 | C2 | B1 | B2 | C1 | A1 | A2 |
|  | 7 | A1 | B2 | B1 | A2 | C1 | C2 |
|  | 8 | B2 | B1 | C2 | C1 | A2 | A1 |
|  | 9 | B2 | A2 | C1 | A1 | C2 | B1 |
|  | 10 | B1 | B2 | C1 | C2 | A1 | A2 |
| Non-Repeating Metrical Patterns | 11 | B1 | A2 | A1 | C2 | B2 | C1 |
|  | 12 | A2 | C1 | B1 | A1 | C2 | B2 |
|  | 13 | C1 | C2 | A2 | B2 | A1 | B1 |
|  | 14 | B2 | A1 | C2 | C1 | B1 | A2 |
|  | 15 | C2 | B2 | C1 | B1 | A2 | A1 |

#### Appendix B – The six word-sets used in Experiment 1

|  |  | Words-sets by Metrical Pattern | | | | | |
| --- | --- | --- | --- | --- | --- | --- | --- |
|  |  | A1 | A2 | B1 | B2 | C1 | C2 |
| Words | 1 | AID | BEACH | ARRANGMENT | ATTITUDE | AUTHORITY | COMEPETITION |
|  | 2 | BLUE | CAMP | CONDITION | CENTURY | AVAILABLE | DEMOCRATIC |
|  | 3 | DANCE | DESK | CONSDIER | CONFIDENCE | CAPACITY | EDUCATION |
|  | 4 | EAST | FILE | DISTINCTION | GENERALLY | ESPCIALLY | OBSERVATION |
|  | 5 | GREEN | HARD | ELECTRIC | MEDICAL | IMPOSSIBLE | PREPARATION |
|  | 6 | KING | LOCK | ESTABLISH | NEWSPAPAER | PARTICULAR | RADIATION |
|  | 7 | MOON | NOSE | FOUNDATION | POPULAR | PHILOSOPHY | RESOLUTION |
|  | 8 | OUT | RAIN | INSTRUCTION | SENSITIVE | REALITY | SCIENTIFIC |
|  | 9 | SEND | TASTE | LOCATION | TELEPHONE | SIGNIFICANTLY | UNDERSTANDING |
|  | 10 | TOWN | WAIT | POSITION | UNIVERSE | SOCIETY | - |

#### Appendix C – Additional Analysis from Experiment 1

###### Model 2

Random effects, fixed effect, and outcome variable

A further analysis was suggested in our preregistration, and so we include it for completeness: if filler-position was not significant in Model 1, which it was not, then we said we would look at the difference between the *repeating metrical pattern list* and the *non-repeating metrical pattern list* at whatever level of *Position Type* that difference was biggest. The biggest difference is when the *non-repeating metrical list* occurs in the final position of a block. The outcome variable, as in Model 1, is whether or not the participant recalled the correct item in the correct position. The model, presented in the popular lme 4 syntax, was as follows:

Error ~ pattern_type + (pattern_type|subject) + (pattern_type|metrical_pattern)

Results of Model 2

The main effect of *Pattern Type* was significant: z=-4.83, p < 0.001, replicating the finding from Model 1 that there does indeed appear to be a performance difference between the last *repeating metrical pattern list* and the adjacent *non-repeating metrical pattern list.* This is strong evidence that individuals have learnt some representational information relating to the *metrical pattern* underlying the *repeating metrical pattern* lists.

#### Appendix D – Additional Analysis of the learning gradients for Experiment 2, 3, and 4

One of the Reviewers suggested it might be beneficial to show the learning gradients in Experiment 2, 3, and 4, comparing a sequence of lists with a Repeated Metrical Pattern, with a similar sequence of Nonrepeating Lists. Clearly, this was not part of our preregistered analysis plan. In that plan, we preferred to look at the performance on specific lists (e.g., the last in a repeating block vs. the first non-repeating list that follows). We believed that that was a more appropriate measure, given that we knew little about the learning gradients that we might expect. Nonetheless, here we present an analysis in line with Reviewer’s suggestion, whereby we take the first three repeating lists in a given repeating block, and compare performance on them with that on the first three non-repeating lists in a subsequent non-repeating block. We chose to look at the gradient across three lists in each case, because that is the maximum number of non-repeating lists that is present in all three experiments of interest (Exps. 2, 3, and 4), and is also the place where we see the clearest evidence of “metrical learning”.

Using GLMMs, we created a model for each experiment, where the two fixed effects were (1) list type, which was categorical, with two levels: (i) repeating, and (ii) non-repeating; and (2) trial, which was continuous and centred. Looking at the interaction term between these two variables reveals whether the learning gradient across trial was reliably different for repeating-metrical-pattern vs non-repeating lists. All tests revealed a reliable interaction term (Exp 2: p=0.021, Exp 3: p=0.041, Exp 4: p=0.003), indicating that in each experiment the gradient of error reduction was significantly steeper in the repeating-metrical-pattern-list condition than in the non-repeating list condition. The data are plotted in Fig 21 below.


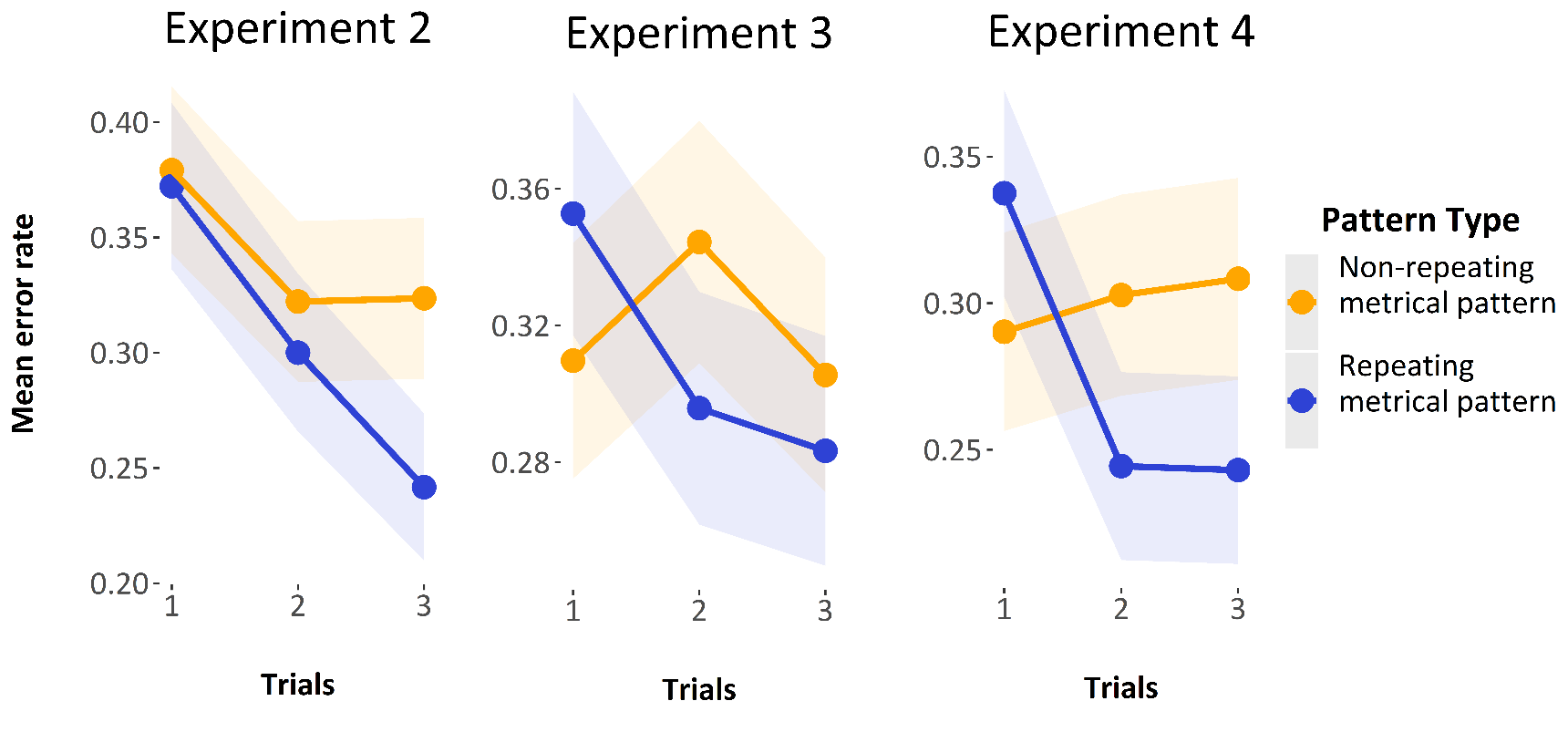


Figure 21. The mean error rate over the first three repeating and non-repeating trials for Experiment 2, 3 and 4. Errors bars show 2 standard errors computed on the total observations.
